# Genetic overlap between psychotic experiences in the community across age and with psychiatric disorders

**DOI:** 10.1038/s41398-020-0765-2

**Published:** 2020-03-09

**Authors:** Wikus Barkhuizen, Oliver Pain, Frank Dudbridge, Angelica Ronald

**Affiliations:** 1 Centre for Brain and Cognitive Development, Department of Psychological Sciences, Birkbeck, University of London, London, UK; 2 NIHR Maudsley Biomedical Research Centre, Social, Genetic and Developmental Psychiatry Centre, Institute of Psychiatry, Psychology and Neuroscience, King’s College London, London, UK; 3 Department of Health Sciences, University of Leicester, Leicester, UK

**Keywords:** Genomics, Psychiatric disorders

## Abstract

This study explores the degree to which genetic influences on psychotic experiences are stable across adolescence and adulthood, and their overlap with psychiatric disorders. Genome-wide association results were obtained for adolescent psychotic experiences and negative symptom traits (*N* = 6297–10,098), schizotypy (*N* = 3967–4057) and positive psychotic experiences in adulthood (*N* = 116,787–117,794), schizophrenia (*N* = 150,064), bipolar disorder (*N* = 41,653), and depression (*N* = 173,005). Linkage disequilibrium score regression was used to estimate genetic correlations. Implicated genes from functional and gene-based analyses were compared. Mendelian randomization was performed on trait pairs with significant genetic correlations. Results indicated that subclinical auditory and visual hallucinations and delusions of persecution during adulthood were significantly genetically correlated with schizophrenia (*r*_g_ = 0.27–0.67) and major depression (*r*_g_ = 0.41–96) after correction for multiple testing. Auditory and visual subclinical hallucinations were highly genetically correlated (*r*_g_ = 0.95). Cross-age genetic correlations for psychotic experiences were not significant. Gene mapping and association analyses revealed 14 possible genes associated with psychotic experiences that overlapped across age for psychotic experiences or between psychotic experiences and psychiatric disorders. Mendelian randomization indicated bidirectional associations between auditory and visual hallucinations in adults but did not support causal relationships between psychotic experiences and psychiatric disorders. These findings indicate that psychotic experiences in adulthood may be more linked genetically to schizophrenia and major depression than psychotic experiences in adolescence. Our study implicated specific genes that are associated with psychotic experiences across development, as well as genes shared between psychotic experiences and psychiatric disorders.

## Introduction

Psychotic experiences (also called “psychotic-like experiences”) resemble positive symptoms of psychotic disorders, such as paranoia, hallucinations, and cognitive symptoms, and have a median prevalence rate of ~7%–8% in the general population^1,2^. Negative symptom traits in the community resemble negative symptoms of psychotic disorders such as apathy, anhedonia, and social withdrawal. Schizotypy^3^ is a related, older personality-based construct compared with psychotic experiences and negative symptoms (PENS). Positive psychotic experiences during adolescence or adulthood, especially when persistent, are associated with an increased risk of developing psychotic disorders^4–9^ and, to a lesser extent, with other psychiatric disorders^10–12^. Likewise, schizotypy is associated with subsequently developing psychotic disorders^13^.

Twin studies suggest heritability accounts for a third to a half of variation in PENS during adolescence^14–19^. Genome-wide association studies (GWAS) indicate modest single-nucleotide polymorphism heritability (SNP-h^2^) for some PENS in mid-adolescence (3%–9%) and for schizotypy in adults (20%–27%)^20–22^. Psychotic experiences share genome-wide genetic influences with schizophrenia and major depression^20,23–26^, although not all studies found this, particularly those that used comparatively smaller samples or polygenic scores (PGS) from less well-powered GWAS^23,24,27–31^. Schizophrenia PGS has been associated with schizotypy in adults assessed using semi-structured interviews, but not with self-rated PENS^32^. Studies have mainly focussed on adolescents and young adults rather than older adults and have not reported on the genetic overlap of psychotic experiences across age, despite this being an important topic for understanding the etiology and development of mental illness. It is also not known whether psychotic experiences and schizotypy share genetic influences.

This study investigates the genetic overlap between psychotic experiences across age using the largest current GWAS summary statistics available for adolescent PENS and for schizotypy and positive psychotic experiences measured in adulthood. We evaluate genome-wide genetic correlations and overlapping associated genes between these trait measures and with schizophrenia, depression, and bipolar disorder. For traits and disorders that share common additive genetic influences, we further explore the nature of these associations using Mendelian randomization (MR).

## Methods and materials

### Samples and measures

#### PENS traits during adolescence

Summary statistics for adolescent PENS came from a mega-GWAS of three European community samples (*N* = 6297–10,098)^20^: Twins Early Developmental Study (TEDS)^33^, a community sample born between 1994–1996 in England and Wales (mean age 16.32 years); Avon Longitudinal Study of Parents and Children (ALSPAC)^34,35^, a birth cohort from the United Kingdom (mean age 16.76 years) born in 1991–1992; and Child and Adolescent Twin Study in Sweden (CATSS)^36^ that recruited twins born in Sweden since 1992 (mean age 18.31 years).

In TEDS, PENS items came from the Specific Psychotic Experiences Questionnaire^37^ and were matched by a team of clinicians to similar items from psychopathology questionnaires available in CATSS and ALSPAC^20^. After harmonization, PENS included four continuous subscales that assessed the frequency or severity of paranoia and hallucinations, cognitive disorganization, anhedonia, and parent-rated negative symptoms.

#### Schizotypy during adulthood

Schizotypy was assessed in the Northern Finland Birth Cohort 1996 (NFBC)^38^ when participants were aged 31 years. GWAS summary statistics^22^ of four continuous schizotypy scales were included (*N* = 3967–4057): perceptual aberrations were assessed with the Perceptual Aberration Scale^39^ and included experiences that resemble clinical features of schizophrenia. Hypomania was from the Hypomanic Personality Scale^40^, devised to assess hypomania, gregariousness, grandiosity, and euphoria. Two scales from Chapman’s Schizotypia Scales were employed: the Revised Social Anhedonia Scale and the Revised Physical Anhedonia Scale^41^, devised to assess the inability to take pleasure from physical and social stimuli, respectively.

#### Positive psychotic experiences assessed in adults

GWAS summary statistics of four dichotomous items from the UK Biobank were obtained from Neale Lab (http://www.nealelab.is/uk-biobank*)* for individuals of European ancestry (*N* = 116,787–117,794). Items assessed psychotic experiences in adults aged 40–69 years: whether participants ever experienced auditory hallucinations, visual hallucinations, delusions of persecution, and delusions of reference.

Additional information on psychotic experiences items are provided in the Supplementary Note.

#### Psychiatric disorders

GWAS summary statistics for schizophrenia^42^, bipolar disorder^43^, and major depressive disorder^44^ were downloaded from the Psychiatric Genetics Consortium (https://www.med.unc.edu/pgc/results-and-downloads). Summary statistics for depression excluded 23andMe participants.

### Analyses

Quality-control procedures were applied to summary statistics prior to analyses. Genetic variants were removed if they had incomplete association statistics, were non-biallelic, or strand ambiguous. Variants with alleles that did not match those in the 1000 Genomes reference panel (phase 3), with info scores < 0.9 and minor allele frequency < 0.01 were excluded.

Linkage disequilibrium (LD) score regression^45^ was used to estimate SNP-h^2^ for traits and the genetic correlation (*r*_g_) between traits. Variants were merged with the HapMap3 reference panel to ensure good imputation quality^45^. Heritability and genetic correlations were converted to liability scales using lifetime prevalence of 1% for schizophrenia, 15% for depression, and 2% for bipolar disorder^46–48^. Effective sample sizes were used for adolescent PENS, because TEDS and CATSS included siblings^20^. To account for sample overlap, the LD score regression intercept was left unconstrained to estimate associations between positive psychotic experiences and depression, because both included UK Biobank participants, and between psychiatric disorders that include PGC participants. Benjamini–Hochberg correction for multiple testing was performed at a false discovery rate of 0.05 to account for 105 correlations estimated.

Gene-wide associations and gene mapping were performed using the FUMA pipeline^49^ and results were compared across psychotic experiences and psychiatric disorders. To identify genes at genome-wide significance, MAGMA v1.06^50^ was used to aggregate the *p*-values of SNPs within gene coding regions (0 kb annotation window). Default parameters and an SNP-wide mean model were employed. Bonferroni-corrected *p*-value thresholds were set for the number of genes tested within each phenotype. Positional mapping of variants within 10 kb of gene regions and likely to have functional consequences (Combined Annotation-Dependent Depletion score (CADD score) ≥ 12.37), gene mapping using expression quantitative trait loci associations and chromatin interactions were performed for independent lead SNPs (at *p* < 1 × 10^–5^ for psychotic experiences, *p* < 1 × 10^−6^ for depression, and *p* < 1 × 10^−8^ for schizophrenia and bipolar disorder) and 1000 Genomes reference panel variants in LD with independent SNPs at *r*^2^ ≥ 0.6 using recommended parameters^49^. Additional details are provided in the Supplementary Note.

MR^51^ was conducted to test for causal relationships between phenotypes that had significant genetic correlations. SNPs were selected as instrumental variables based on the clumping algorithm in PLINK^52^ using an *r*^2^ threshold of 0.05 within a 500 kb window at genome-wide significant levels (*p* < 5 × 10^−8^) for schizophrenia. Summary statistics for depression did not include 23andMe participants and had an insufficient number of genome-wide significant variants for MR analyses. Instead, genome-wide significant variants were obtained from the publication^44^. Insufficient genome-wide significant variants associated with psychotic experiences meant that *p*-value thresholds for psychotic experiences were set at *p* < 5 × 10^−5^ to allow for at least 20 instrumental variables. We report MR analyses using equivalent *p*-value thresholds for all phenotypes (*p* < 5 × 10^−5^) in the Supplementary Material.

Generalized summary-data-based MR (GSMR) was used due to its advantages of accounting for residual LD structure between instrumental variables (set at LD *r*^2^ > 0.1) and for sampling variation in the exposure and outcome GWAS^53^. MR-Egger regression^54^, weighted median^55^, and weighted mode^56^ methods were conducted as sensitivity analyses for possible violation of MR assumptions. SNPs identified as potentially pleiotropic or with residual LD structure in GSMR Heidi-outlier analyses were also excluded from sensitivity analyses.

## Results

Common additive genetic variance accounted for 8%–10% of phenotypic variation in adolescent PENS (paranoia/hallucinations and negative symptoms did not have significant SNP-h^2^ estimates in these analyses), 30%–37% in schizotypy during adulthood, and 7%–10% in positive psychotic experiences during adulthood (Table 1).

**Table 1:** Genome-wide association study sample sizes and SNP-heritability estimates.

	GWAS *N*	*N* cases	QC-positive SNPs	SNP-h^2^	SE	*p*
Adolescent psychotic experiences and negative symptom traits						
Paranoia and hallucinations	8665	Continuous	3,363,829	−0.0042	0.0352	0.453
Cognitive disorganization	6297	Continuous	3,363,829	0.1048	0.0566	0.032
Anhedonia	6579	Continuous	3,363,829	0.0797	0.0479	0.048
Parent-rated negative symptoms	10,098	Continuous	3,363,829	−0.0222	0.0316	0.241
Schizotypy during adulthood						
Hypomania	3967	Continuous	5,493,986	0.3732	0.1011	<0.001
Perceptual aberrations	4057	Continuous	5,493,986	0.3037	0.0916	<0.001
Physical anhedonia	3988	Continuous	5,493,986	0.3655	0.0965	<0.001
Social anhedonia	4025	Continuous	5,493,986	0.2950	0.0826	<0.001
Positive psychotic experiences during adulthood						
Auditory hallucinations	117,503	2009	6,443,634	0.0709	0.0255	0.003
Visual hallucinations	116,787	3768	6,443,706	0.1032	0.0224	<0.001
Delusions of persecution	117,794	932	6,443,695	0.0910	0.0521	0.040
Delusions of reference	117,731	822	6,443,693	0.0666	0.0499	0.091
Psychiatric disorders						
Schizophrenia	150,064	36,989	5,274,747	0.1631	0.0044	<0.001
Bipolar disorder	41,653	20,129	5,083,505	0.2999	0.0102	<0.001
Major Depression	173,005	59,851	5,488,968	0.0999	0.0042	<0.001

*SNP-h*^*2*^ univariate SNP heritability. SNP-h^2^ converted to a liability scale for binary traits; effective sample size used for adolescent PENS in LD score regression analyses to account for the presence of siblings (paranoia and hallucinations = 7970.416; cognitive disorganization = 5082.760; anhedonia = 6068.311; parent-rated negative symptoms = 8763.295).

**Fig. 1:**
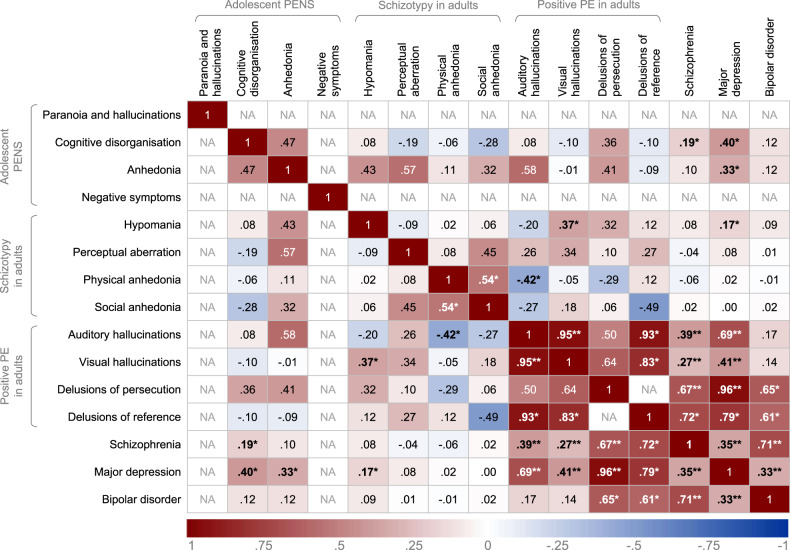
Heat map showing genetic correlations between psychotic experiences and psychiatric disorders. PENS, Psychotic experiences (PE) and negative symptom traits; NA, genetic correlations could not be computed due to low SNP heritability or sample size (see Supplementary Table S1 for genetic covariance estimates). *Nominally statistically significant genetic correlations at *p* < .05; **genetic correlations that survived Benjamini–Hochberg correction for multiple testing for 105 pairwise correlations (at a FDR of 0.05); genetic correlations reported using unconstrained LD score regression intercept between phenotypes with sample overlap.

Genetic correlations are shown in Fig. 1. Genetic correlations could not be computed for paranoia and hallucinations, and negative symptoms, likely due to low SNP-h^2^. For these comparisons, we report genetic covariance (*ρ*_g_), which indicates direction of correlation (Supplementary Table S1).

### Genetic correlations with psychiatric disorders

For positive psychotic experiences in UK Biobank, auditory hallucinations were significantly genetically correlated with schizophrenia (*r*_g_ = 0.39, *p* = 2.27 × 10^−5^) and depression (*r*_g_ = 0.69, *p* = 8.07 × 10^−6^), but not with bipolar disorder (*r*_g_ = 0.17, *p* = 0.082). Likewise, visual hallucinations was significantly correlated with schizophrenia (*r*_g_ = 0.27, *p* = 4.12 × 10^−7^) and depression (*r*_g_ = 0.41, *p* = 2.00 × 10^−4^) but not with bipolar disorder (*r*_g_ = 0.14, *p* = 0.149). We observed high and significant genetic correlations between delusions of persecution with schizophrenia (*r*_g_ = 0.67, *p* = 0.002) and depression (*r*_g_ = 0.96, *p* = 0.001), whereas the genetic correlation with bipolar disorder was not significant after correction for multiple testing (*r*_g_ = 0.65, *p* = .019). Genetic correlations between delusions of reference with schizophrenia (*r*_g_ = 0.72, *p* = 0.007), depression (*r*_g_ = 0.79, *p* = 0.021) and bipolar disorder (*r*_g_ = 0.61, *p* = 0.033) were at nominal significance (*p* < 0.05) but did not survive correction for multiple testing.

Between adolescent PENS and psychiatric disorders, genetic correlations were not significant after correction for multiple testing. Nominally significant genetic correlations were observed between cognitive disorganization with schizophrenia (*r*_g_ = 0.19, *p* = 0.034) and depression (*r*_g_ = 0.40, *p* = 0.006) and between anhedonia and depression (*r*_g_ = 0.33, *p* = 0.021). Negative symptoms covaried positively with schizophrenia (*ρ*_g_ = 0.03, *p* = 1.37 × 10^−4^) and depression (*ρ*_g_ = 0.03, *p* = 2.41 × 10^−7^). Positive genetic covariation was observed between paranoia and hallucinations, and depression (*ρ*_g_ = 0.03, *p* = 1.43 × 10^−4^). No significant genetic correlations were found with bipolar disorder.

Between psychiatric disorders and schizotypy in adults, hypomania was genetically correlated with depression at nominal significance (*r*_g_ = 0.17, *p* = 0.014). We did not find evidence that the schizotypy scales correlated with schizophrenia or bipolar disorder.

### Genetic stability of psychotic experiences across age

PENS during adolescence were not significantly genetically correlated with positive psychotic experiences and schizotypy during adulthood. Between the adult samples, hypomania was genetically correlated with visual hallucinations (*r*_g_ = 0.37, *p* = 0.024). Physical anhedonia showed a negative genetic correlation with auditory hallucinations (*r*_g_ = −0.42, *p* = 0.034), but not after correction for multiple testing.

### Genetic correlations within samples

Within positive psychotic experiences in UK Biobank, auditory and visual hallucinations were highly and significantly genetically correlated (*r*_g_ = 0.95, *p* = 7.61 × 10^−5^). Genetic correlations between delusions of reference with auditory (*r*_g_ = 0.93, *p* = 0.038) and visual hallucinations (*r*_g_ = 0.83, *p* = 0.050) were not significant after correction for multiple testing. Delusions of persecution were not genetically correlated with auditory or visual hallucinations. A genetic correlation between delusions of persecution and delusions of reference could not be computed due to low SNP-h^2^ in both phenotypes. but these two items showed positive genetic covariance (*ρ*_g_ = 0.01, *p* = 0.003).

There was a nominally significant association between physical and social anhedonia (*r*_g_ = 0.54, *p* = 0.044). No other genetic correlations between schizotypy scales were significant.

Within adolescent PENS scales, positive genetic covariance was identified between anhedonia and negative symptoms (*ρ*_g_ = 0.11, *p* = 0.012). No significant genetic overlap between other adolescent PENS was found.

### Comparison of implicated genes across mapping strategies and phenotypes

**Fig. 2:**
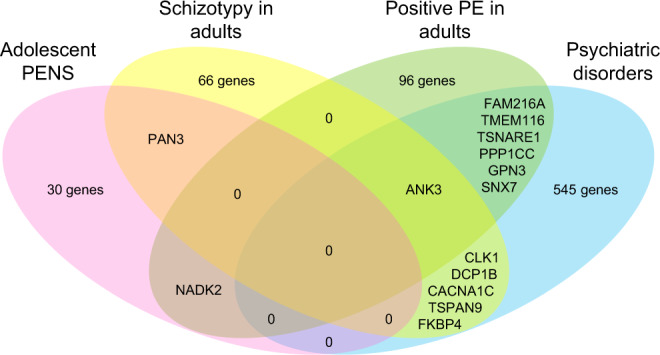
Number of overlapping genes between psychotic experiences across age and psychiatric disorders. PENS, Psychotic experiences (PE) and negative symptom traits. Genes identified using (**a**) genome-wide gene associations in MAGMA after Bonferroni correction for the number of gene associations tested, (**b**) positional mapping that prioritized genes based on variant functional annotations obtained using ANNOVAR, (**c**) eQTL (expressive quantitative trait) mapping, and (**d**) chromatin interaction mapping.

Gene mapping and genome-wide gene association results from FUMA were compared across psychotic experiences and psychiatric disorders (Table 2). Full results for each phenotype are provided in Supplementary Figs. S1–3 and Supplementary Tables S2–7. Results revealed 32 genes for adolescent PENS, of which *PAN3* mapped to adolescent cognitive disorganization and schizotypy in adulthood (perceptual aberrations), and *NADK2* to adolescent negative symptoms and delusions of reference in UK Biobank; none overlapped with psychiatric disorders. Seventy-three genes were found for adult schizotypy including the aforementioned *PAN3* and six genes that were also indicated for schizophrenia. *ANK3* overlapped with both positive psychotic experiences (visual hallucinations) and schizophrenia. For positive psychotic experiences in adults, 104 genes were identified, seven of which overlapped with schizophrenia and, as mentioned above, one with schizotypy and one with adolescent PENS (Fig. 2).

**Table 2:** Gene-associations and gene-mapping results for overlapping genes across phenotypes.

Gene	Chr	Phenotype	MAGMA		Positional mapping		eQTL mapping I			Chromatin Interactions	Mapped SNPs min *p*_GWAS_	Independent lead SNPs
			*n* SNPs	*p*_MAGMA_	*n* SNPs	*CADD*	*n* SNPs	*GTEx map*	*Min p*_eQTL_	*HiCi cortex*		
*ANK3*	10	Physical anhedonia	-	-	4	15.60	-	-	-	-	6.35 × 10^−6^	rs72818480
		Unreal visions	-	-	1	13.71	-	-	-	-	9.71 × 10^−6^	rs10994279
		Schizophrenia	1580	9.46 × 10^−7^	-	-	-	-	-	-	-	-
*CACNA1C*	12	Hypomania	-	-	3	17.74	85	CerHem; Cereb	7.34 × 10^−9^	-	1.91 × 10^−6^	rs6489351; rs3922316; rs34382810
		Schizophrenia	1295	7.79 × 10^−20^	4	17.74	86	CerHem; Cereb	7.34 × 10^−9^	-	2.63 × 10^−17^	rs6489351; rs11062159; rs12423277; rs2239037; rs2007044; rs882193; rs4765914; rs2239063
*CLK1*	2	Perceptual aberrations	-	-	-	-	-	-	-	Fetal	4.65 × 10^−6^	rs56225831
		Schizophrenia	-	-	-	-	-	-	-	Adult	3.60 × 10^−13^	rs796364; rs74938253
*DCP1B*	12	Hypomania	-	-	-	-	-	-	-	Fetal/adult	2.16 × 10^−6^	rs34382810; rs3922316; rs6489351
		Schizophrenia	-	-	-	-	-	-	-	Fetal/adult	1.65 × 10^−16^	rs2007044; rs11062159; rs2239037; rs12423277; rs6489351; rs2239063
*FAM216A*	12	Unreal visions	-	-	-	-	-	-	-	Fetal	1.55 × 10^−6^	rs4766567
		Schizophrenia	-	-	-	-	-	-	-	Fetal	7.09 × 10^−10^	rs4766428
*FKBP4*	12	Hypomania	-	-	-	-	-	-	-	Fetal/adult	2.16 × 10^−6^	rs34382810; rs3922316
		Schizophrenia	-	-	-	-	-	-	-	Fetal/adult	1.65 × 10^−16^	rs2007044; rs12423277; rs11062159; rs2239037
*GPN3*	12	Unreal visions	-	-	-	-	-	-	-	Fetal	1.55 × 10^−6^	rs4766567
		Schizophrenia	-	-	-	-	-	-	-	Fetal	7.09 × 10^−10^	rs4766428
*NADK2*	5	Negative symptoms	-	-	-	-	-	-	-	Fetal/adult	NA^a^	rs147205145
		Unreal communications	-	-	-	-	-	-	-	Adult	3.68 × 10^−6^	rs16902775
*PAN3*	13	Cognitive disorganization	-	-	1	14.24	-	-	-	-	NA^a^	rs9513058
		Perceptual aberrations	-	-	-	-	-	-	-	Adult	4.92 × 10^−3^	rs9581857
*PPP1CC*	12	Unreal visions	-	-	-	-	-	-	-	Fetal	1.55 × 10^−6^	rs4766567
		Schizophrenia	-	-	-	-	-	-	-	Fetal	7.09 × 10^−10^	rs4766428
*SNX7*	1	Unreal visions	-	-	-	-	-	-	-	Fetal	3.78 × 10^−4^	rs34186519
		Unreal conspiracy	-	-	-	-	-	-	-	Fetal	4.42 × 10^−8^	rs4908317; rs17403033
		Schizophrenia	-	-	-	-	-	-	-	Fetal/adult	2.83 × 10^−17^	rs1702294; rs2802525; rs61786697; rs7526108
*TMEM116*	12	Unreal visions	-	-	-	-	2	BasalG	7.86 × 10^−7^	-	1.76 ×10^−5^	rs4766567
		Schizophrenia	-	-	-	-	-	-	-	Fetal	7.09 × 10^−10^	rs4766428
*TSNARE1*	8	Unreal communications	-	-	-	-	3	CerHem	7.77 × 10^−7^	Fetal/adult	5.64 × 10^−5^	rs10102944
		Schizophrenia	502	5.26 ×10^-10^	1	13.06	-	-	-	Fetal/adult	2.85 × 10^−13^	rs4129585; rs74576444; rs62512616; rs13282237; rs72687362
*TSPAN9*	12	Hypomania	-	-	-	-	-	-	-	Fetal/adult	2.16 × 10^−6^	rs34382810; rs3922316
		Schizophrenia	-	-	-	-	-	-	-	Fetal/adult	1.65 × 10^−16^	rs2007044; rs12423277; rs11062159; rs2239037; rs2239063

*BasalG* GTEx v6 Basal ganglia tissue map, *CADD* The maximum Combined Annotation-Dependent Depletion score, *Cereb* GTEx v7 cerebellum tissue map, *CerHem* GTEx v7 Cerebellar hemisphere tissue map, *Chr* chromosome number, *Min pGWAS* minimum *p*-value of annotated or mapped variants from summary statistics. SNPs mapped to genes included independent lead SNPs and SNPs from 1K Genomes reference panel in LD with lead SNPs (*r*^2^ ≥ 0.6), within 10 kb of locus and likely to be deleterious (CADD ≥ 12.37).

^a^Annotated or mapped variants present only in reference panel and therefore does not have a minimum pGWAS.

### Mendelian randomization

Results from GSMR analyses and MR sensitivity analyses are summarized in Table 3, and MR-Egger intercept tests, heterogeneity statistics, and plots in Supplementary Table S8 and Supplementary Figs. S4–5.

**Table 3:** Mendelian randomization analyses.

Exposure		Outcome	Heidi SNPs	LD SNPs	*n* SNP	GSMR results			MR-Egger			Weighted median			Weighted mode		
						*β*	SE	*p*	*β*	SE	*p*	*β*	SE	*p*	*β*	SE	*p*
Auditory hallucinations	**→**	Schizophrenia	0	0	49	0.021	0.676	0.975	2.494	1.996	0.218	−0.008	0.908	0.993	−0.216	1.993	0.914
Schizophrenia	**→**	Auditory hallucinations	0	11	98	0.001	0.001	0.281	0.001	0.004	0.846	0.001	0.001	0.424	0.003	0.003	0.281
Visual hallucinations	**→**	Schizophrenia	0	0	48	−0.115	0.501	0.819	−1.860	1.697	0.279	−0.132	0.707	0.852	−1.091	1.635	0.508
Schizophrenia	**→**	Visual hallucinations	2	11	96	0.002	0.001	0.084	−0.003	0.005	0.514	0.003	0.002	0.099	0.005	0.004	0.270
Auditory hallucinations	**→**	Major depression	0	0	55	−0.269	0.488	0.581	1.025	1.405	0.469	0.091	0.685	0.895	0.502	1.626	0.759
Major depression	**→**	Auditory hallucinations	0	0	36	0.000	0.000	0.424	0.007	0.003	**0.042**	0.000	0.000	0.081	0.000	0.000	0.140
Auditory hallucinations	**→**	Visual hallucinations	0	0	97	0.321	0.034	**1.73 ×10**^**−21**^	0.310	0.071	**3.47 ×10**^**−5**^	0.333	0.046	**5.16 ×10**^**−13**^	0.505	0.133	**2.66 ×10**^**−4**^
Visual hallucinations	**→**	Auditory hallucinations	0	0	85	0.166	0.019	**4.25 ×10**^**−18**^	0.123	0.043	**0.005**	0.168	0.026	**1.38 ×10**^**−10**^	0.211	0.065	**1.62 ×10**^**−3**^
Visual hallucinations	**→**	Major depression	0	0	52	0.263	0.362	0.468	−0.711	1.036	0.496	0.337	0.524	0.520	0.376	1.253	0.765
Major depression	**→**	Visual hallucinations	0	0	36	0.000	0.000	0.986	0.008	0.005	0.103	0.000	0.000	0.834	0.000	0.000	0.576
Delusions of persecution	**→**	Major depression	0	0	49	0.356	0.742	0.631	−1.264	2.031	0.537	1.211	1.088	0.266	1.780	2.381	0.458
Major depression	**→**	Delusions of persecution	0	0	36	0.000	0.000	0.857	0.007	0.002	**0.003**	0.000	0.000	0.768	0.000	0.000	0.814
Delusions of persecution	**→**	Schizophrenia	0	0	46	−0.020	1.025	0.984	1.976	3.466	0.572	−0.401	1.482	0.787	−0.219	2.835	0.939
Schizophrenia	**→**	Delusions of persecution	1	11	97	0.002	0.001	**9.33 ×10**^**−5**^	0.001	0.003	0.810	0.002	0.001	**0.006**	0.002	0.002	0.397

*GS*_*MR*_ generalized summary-based Mendelian randomization; *LD SNPs* SNPs with residual LD at *r*^2^ > 0.1 removed from analysis; n *SNPs* number of variants remaining in analyses after those identified as Heidi outliers or with residual LD was removed.

SNPs identified as having residual LD and as Heidi outliers were also excluded from MR-Egger, weighted median, and weighted mode analyses.

Bold text indicates significant *p*-values at *p* < 0.05.

GSMR analyses provided evidence for a bidirectional association between auditory and visual hallucinations (*P*_GSMR_ = 1.73 × 10^−21^ for auditory hallucinations as the exposure; *P*_GSMR_ = 4.25 × 10^−25^ for visual hallucinations as the exposure) and replicated in all MR sensitivity analyses.

We observed evidence of a directional effect of schizophrenia liability on delusions of persecution (*P*_GSMR_ = 9.33 × 10^−5^); however, the effect was small and replicated in weighted median but not in MR-Egger, nor in weighted mode analyses. No evidence of a significant effect in the other direction was found.

Only the MR-Egger method indicated significant directional effects of a liability to depression on a propensity to report auditory hallucinations and delusions of persecution. However, MR-Egger intercept tests indicated the presence of directional pleiotropy in both instances (Supplementary Table S8), indicating that MR-Egger results may be reliable as it adjusts for non-zero intercepts allowing for more robust estimates when horizontal pleiotropy is present compared with the other methods. The discrepancy between MR methods may also be due to outliers not identified by Heidi-outlier analysis. MR analyses using IVs selected at *p* < 5 × 10^−5^ for all exposures did not affect our main conclusions (Supplementary Table S9).

## Discussion

This study investigated whether common genetic variation underlying psychotic experiences overlaps across adolescence and adulthood, and with psychiatric disorders. Our results suggest that with increasing age from adolescence to adulthood, psychotic experiences become more linked genetically to schizophrenia and major depression. We did not observe evidence of significant genetic stability of psychotic experiences across adolescence and adulthood in existing data. However, our study implicated specific genes that are associated with psychotic experiences across development and genes shared between psychotic experiences and psychiatric disorders.

### Associations between psychotic experiences and psychiatric disorders

We found that positive psychotic experiences in UK Biobank (specifically experiences of auditory and visual hallucinations) shared a moderate to substantial amount of common genetic variation with depression and schizophrenia, consistent with an independent report^26^. Our MR analyses indicated that liability to schizophrenia may be directionally associated with a propensity to experience delusions of persecution and depression liability with a propensity to report auditory hallucinations and delusions of persecution. This latter finding concurs with a twin study showing that depression and psychotic experiences phenotypically influence one another in adolescence over and above genetic influences^57^. However, these MR effect sizes were small and not consistently replicated in sensitivity analyses, and are therefore unlikely to reflect true causal effects.

This study reported genetic overlap between adolescent PENS and psychiatric disorders. We used more recent summary statistics from larger GWAS of depression and bipolar disorder^43,44^, which allowed more reliable estimations compared with previous studies^20^. Our results add to the growing body of evidence that there are genetic associations between adolescent PENS and psychiatric disorders^20,24^. There were more significant genetic correlations between psychotic experiences and depression than with schizophrenia. This could be explored further in terms of the underlying reasons.

Schizotypy domains did not appear to share common additive genetic influences with schizophrenia nor with bipolar disorder. There was suggestive evidence of genetic overlap between hypomania and depression. Schizotypy has been associated with an increased risk of schizophrenia-spectrum disorders^13^, but longitudinal evidence of an association between schizotypy and subsequently being diagnosed with schizophrenia is limited^58^ and findings inconsistent^59^.

Bipolar disorder does not appear to share genetic overlap with adolescent PENS or adult schizotypy. It is possible that common genetic influences on bipolar disorder, in contrast to those on depression and schizophrenia, are not involved in these earlier adolescent forms of psychopathology. Future studies with better-powered GWAS will be able to test this hypothesis further.

We found evidence that some genes may be involved in both psychotic experiences and psychiatric disorders. Seven genes were implicated in both positive psychotic experiences in UK Biobank and schizophrenia: *ANK3*, a gene in the 10q21.2 region, has previously been associated with bipolar disorder and schizophrenia^60–62^. *TSNARE1* (8q24.3) has previously been implicated in schizophrenia and cognitive function^42,63^. The gene *SNX7* (1p21.3) has known associations with mathematical ability^64^. Three neighboring genes located on band 12q24.11, *FAM216A*, *PPP1CC*, and *GPN3*, and a nearby gene *TMEM116* (12q24.13) have been associated with white blood cell and platelet count, heart rate, and body mass index^65–67^.

Six mapped genes overlapped between adult schizotypy and psychiatric disorders, including *ANK3* discussed above. As noted elsewhere^22^, we found evidence that *CACNA1C* (12p13.33), with well-replicated associations with psychiatric and developmental disorders^42,60,61^, may be involved in hypomania, schizophrenia, and bipolar disorder. Located within the same 12p13.33 region, the genes *DCP1B*, *TSPAN9*, and *FKBP4* also overlapped between hypomania and schizophrenia, and have previously been associated with a range of physical health phenotypes including obesity and waist–hip ratio^66,68^. Findings indicated the possible involvement of *CLK1* (2q33.1) in perceptual aberrations and schizophrenia, a gene previously associated with breast cancer^69^.

Our results suggest that psychotic experiences may share more genome-wide genetic overlap with schizophrenia and major depression in adulthood than adolescence. Participants had narrow age ranges in the adolescent PENS (15–19 years old) and adult schizotypy (all born in the same year) samples, and were assessed on current psychotic experiences, whereas UK Biobank participants had a wider and older age range (40–69 years old), and were asked to report on lifetime psychotic experiences. It is therefore possible that psychotic experiences reported in UK Biobank may have been persistent rather than transitory. According to the persistent-impairment model, those with persistent psychotic experiences are at a higher risk of developing psychotic disorders and may therefore share more genetic influences with psychiatric disorders^70^. It is also possible that some of those in the adolescent samples may be genetically liable to psychotic experiences but will have not yet developed them. A previous study found that genetic liability to schizophrenia was positively associated with adolescent paranoia and hallucinations only after excluding non-zero scores^20^, suggesting that genetic liability to schizophrenia may vary in adolescents who did not (yet) report psychotic experiences. This may explain why we found that genetic overlap between psychotic experiences and psychiatric disorders increased with age.

### Stability of genetic influences on psychotic experiences across age

We did not find significant genetic overlap across PENS (adolescence), schizotypy (adulthood), and positive psychotic experiences (adulthood). Different genetic influences may be involved in psychotic experiences across the lifespan. For example, some psychotic experiences may be adolescent-specific, tied in with development and lifestyle factors such as sleep problems, risk-taking, and experimenting with drugs. Psychotic experiences in older adults may have different etiological pathways, in part associated with conditions such as medication side effects, dementia, eye conditions, and Charles Bonnet syndrome. However, it was not possible to rule out that the different measurements used across ages led to the lack of genetic overlap. Another reason why no cross-age genetic overlap was found may be the smaller GWAS sample sizes for adolescent PENS and adult schizotypy. In agreement with our findings, an independent report found that genetic risk for psychotic experiences in adults did not predict psychotic experiences during mid-adolescence^26^.

We found that some of the same genes may be involved in psychotic experiences across age. The gene *ANK3* was implicated for adult schizotypy and adult psychotic experiences. The gene *PAN3* (13q12.2) was implicated in adolescent cognitive disorganization and schizotypy. *NADK2* (mitochondrial) on 5p13.2 was mapped to both negative symptoms in adolescence and to adult psychotic experiences.

### Within-sample associations between specific types of psychotic experiences

We also investigated within-sample genetic associations and found that auditory and visual hallucinations during adulthood shared the same common genetic influences and were bidirectionally associated in MR analyses. This bidirectional association may indicate a pervasive shared genetic basis between auditory and visual hallucinatory experiences rather than true causal associations, because pleiotropic effects of the genetic instruments through a latent common precursor (which would violate MR assumptions) seem likely. As such, experiences resembling auditory and visual hallucinations may share biological pathways.

### Limitations

We note caution in interpreting MR results that compared within-sample psychotic experiences, because sample overlap could lead to weak instrument bias away from the null^71^. We may not have detected true causal effects due to weak instrument bias in analyses that used psychotic experiences as the exposure, because genetic variants below conventional genome-wide significance levels were used. Future studies could re-evaluate these causal associations once larger GWAS for psychotic experiences become available. Furthermore, future studies with greater power might explore the associations of psychotic experiences with a wider array of phenotypes.

Many of the overlapping genes we report were identified based mainly on chromatin interactions. The chromatin interaction data had a high resolution (10 kb) resulting in more mapped genes and included enhancer–promoter and promoter–promoter interactions only^72^, therefore providing strong hypotheses for variant–gene associations. For psychotic experiences and depression, functional consequences on genes were annotated to variants clumped below conventional genome-wide significance levels. As such, these results will benefit from replication once better-powered GWAS become available.

There are known limitations when using summary statistics from large biobanks including ascertainment bias and the possibility that collider bias may result in overinflated genetic correlations^73,74^. Loss to follow-up in the cohort studies used in GWAS may have an impact on the generalizability of the genetic findings^75^. However, we note that attrition based on phenotypic or genetic risk for psychosis would lead, if anything, to underestimates of the genetic overlap between psychotic experience traits and psychiatric disorders.

### Implications and conclusions

Psychotic experiences during adulthood and adolescence share genetic influences with psychiatric disorders. This study implicated specific genes that may be involved in both psychotic experiences in the community and psychiatric disorders, and genes associated with psychotic experiences across development. Subclinical experiences of auditory and visual hallucinations in adults may have similar biological etiologies. Our findings and other independent reports indicate that psychotic experiences assessed using different measures at different developmental stages may not reflect genetically similar phenomena.

## Supplementary information

Supplementary Figures and Supplementary Note

Supplementary Tables S2-7

## References

[1] Linscott R, Van OsJ (2013). An updated and conservative systematic review and meta-analysis of epidemiological evidence on psychotic experiences in children and adults: on the pathway from proneness to persistence to dimensional expression across mental disorders. Psychol. Med..

[2] Kelleher I (2012). Prevalence of psychotic symptoms in childhood and adolescence: a systematic review and meta-analysis of population-based studies. Psychol. Med..

[3] Meehl PE (1962). Schizotaxia, schizotypy, schizophrenia. Am. Psychol..

[4] Poulton R (2000). Children’s self-reported psychotic symptoms and adult schizophreniform disorder: a 15-year longitudinal study. Arch. Gen. Psychiatry.

[5] Welham J (2009). Emotional and behavioural antecedents of young adults who screen positive for non-affective psychosis: a 21-year birth cohort study. Psychol. Med..

[6] Hanssen M, Bak M, Bijl R, Vollebergh W, van Os J (2005). The incidence and outcome of subclinical psychotic experiences in the general population. Br. J. Clin. Psychol..

[7] Werbeloff N (2012). Self-reported attenuated psychotic symptoms as forerunners of severe mental disorders later in life. Arch. Gen. Psychiatry.

[8] Zammit S (2013). Psychotic experiences and psychotic disorders at age 18 in relation to psychotic experiences at age 12 in a longitudinal population-based cohort study. Am. J. Psychiatry.

[9] Dominguez MD, Wichers M, Lieb R, Wittchen HU, van Os J (2011). Evidence that onset of clinical psychosis is an outcome of progressively more persistent subclinical psychotic experiences: an 8-year cohort study. Schizophr. Bull..

[10] Fisher HL (2013). Specificity of childhood psychotic symptoms for predicting schizophrenia by 38 years of age: a birth cohort study. Psychol. Med..

[11] Kaymaz N (2012). Do subthreshold psychotic experiences predict clinical outcomes in unselected non-help-seeking population-based samples? A systematic review and meta-analysis, enriched with new results. Psychol. Med..

[12] Kelleher I (2012). Clinicopathological significance of psychotic experiences in non-psychotic young people: evidence from four population-based studies. Br. J. Psychiatry.

[13] Debbané M (2015). Developing psychosis and its risk states through the lens of schizotypy. Schizophr. Bull..

[14] Linney YM (2003). A quantitative genetic analysis of schizotypal personality traits. Psychol. Med..

[15] Zavos HM (2014). Consistent etiology of severe, frequent psychotic experiences and milder, less frequent manifestations: a twin study of specific psychotic experiences in adolescence. JAMA Psychiatry.

[16] Polanczyk G (2010). Etiological and clinical features of childhood psychotic symptoms: results from a birth cohort. Arch. Gen. Psychiatry.

[17] Hur YM, Cherny SS, Sham PC (2012). Heritability of hallucinations in adolescent twins. Psychiatry Res..

[18] Wigman JT (2011). A twin study of genetic and environmental determinants of abnormal persistence of psychotic experiences in young adulthood. Am. J. Med. Genet. B Neuropsychiatr. Genet..

[19] Ericson M, Tuvblad C, Raine A, Young-Wolff K, Baker LA (2011). Heritability and longitudinal stability of schizotypal traits during adolescence. Behav. Genet..

[20] Pain O (2018). Genome-wide analysis of adolescent psychotic-like experiences shows genetic overlap with psychiatric disorders. Am. J. Med. Genet. B Neuropsychiatr. Genet..

[21] Sieradzka D (2015). Heritability of individual psychotic experiences captured by common genetic variants in a community sample of adolescents. Behav. Genet..

[22] Ortega-Alonso A (2017). Genome-wide association study of psychosis proneness in the Finnish population. Schizophr. Bull..

[23] Ronald A, Pain O (2018). A systematic review of genome-wide research on psychotic experiences and negative symptom traits: new revelations and implications for psychiatry. Hum. Mol. Genet..

[24] Jones HJ (2016). Phenotypic manifestation of genetic risk for schizophrenia during adolescence in the general population. JAMA psychiatry.

[25] Taylor MJ (2019). Association of genetic risk factors for psychiatric disorders and traits of these disorders in a Swedish population twin sample. *JAMA*. Psychiatry.

[26] Legge S. E. et al. Association of genetic liability to psychotic experiences with neuropsychotic disorders and traits. *JAMA Psychiatry*10.1001/jamapsychiatry.2019.2508 (2019). 10.1001/jamapsychiatry.2019.2508 PMC6764002 31553412

[27] Zammit S (2014). A population-based study of genetic variation and psychotic experiences in adolescents. Schizophr. Bull..

[28] Sieradzka D (2014). Are genetic risk factors for psychosis also associated with dimension-specific psychotic experiences in adolescence?. PLoS ONE.

[29] Krapohl E (2016). Phenome-wide analysis of genome-wide polygenic scores. Mol. Psychiatry.

[30] Derks EM, Vorstman JA, Ripke S, Kahn RS, Ophoff RA (2012). Investigation of the genetic association between quantitative measures of psychosis and schizophrenia: a polygenic risk score analysis. PLoS ONE.

[31] Liuhanen J (2018). Interaction between compound genetic risk for schizophrenia and high birth weight contributes to social anhedonia and schizophrenia in women. Psychiatry Res..

[32] van Os J (2017). Evidence that polygenic risk for psychotic disorder is expressed in the domain of neurodevelopment, emotion regulation and attribution of salience. Psychol. Med..

[33] Haworth CM, Davis OS, Plomin R (2013). Twins Early Development Study (TEDS): a genetically sensitive investigation of cognitive and behavioral development from childhood to young adulthood. Twin Res. Hum. Genet..

[34] Boyd A (2013). Cohort profile: the ‘children of the 90s’–the index offspring of the Avon Longitudinal Study of Parents and Children. Int. J. Epidemiol..

[35] Fraser A (2013). Cohort profile: the Avon Longitudinal Study of Parents and Children: ALSPAC mothers cohort. Int. J. Epidemiol..

[36] Anckarsater H (2011). The Child and Adolescent Twin Study in Sweden (CATSS). Twin Res. Hum. Genet..

[37] Ronald A (2014). Characterization of psychotic experiences in adolescence using the specific psychotic experiences questionnaire: findings from a study of 5000 16-year-old twins. Schizophr. Bull..

[38] Haapea M (2008). Non-participation in a field survey with respect to psychiatric disorders. Scand. J. Public Health.

[39] Chapman LJ, Chapman JP, Raulin ML (1978). Body-image aberration in Schizophrenia. J. Abnorm. Psychol..

[40] Eckblad M, Chapman LJ (1986). Development and validation of a scale for hypomanic personality. J. Abnorm. Psychol..

[41] Chapman LJ, Chapman JP, Raulin ML (1976). Scales for physical and social anhedonia. J. Abnorm. Psychol..

[42] Schizophrenia Working Group of the Psychiatric Genomics Consortium. (2014). Biological insights from 108 schizophrenia-associated genetic loci. Nature.

[43] Bipolar Disorder and Schizophrenia Working Group of the Psychiatric Genomics Consortium. (2018). Genomic dissection of bipolar disorder and schizophrenia, including 28 subphenotypes. Cell.

[44] Wray NR (2018). Genome-wide association analyses identify 44 risk variants and refine the genetic architecture of major depression. Nat. Genet..

[45] Bulik-Sullivan B (2015). An atlas of genetic correlations across human diseases and traits. Nat. Genet..

[46] Merikangas KR (2011). Prevalence and correlates of bipolar spectrum disorder in the world mental health survey initiative. Arch. Gen. Psychiatry.

[47] Moreno-Kustner B, Martin C, Pastor L (2018). Prevalence of psychotic disorders and its association with methodological issues. A systematic review and meta-analyses. PLoS ONE.

[48] Lim GY (2018). Prevalence of depression in the community from 30 countries between 1994 and 2014. Sci. Rep..

[49] Watanabe K, Taskesen E, van Bochoven A, Posthuma D (2017). Functional mapping and annotation of genetic associations with FUMA. Nat. Commun..

[50] de Leeuw CA, Mooij JM, Heskes T, Posthuma D (2015). MAGMA: generalized gene-set analysis of GWAS data. PLoS Comput. Biol..

[51] Davey Smith G, Ebrahim S (2003). ‘Mendelian randomization’: can genetic epidemiology contribute to understanding environmental determinants of disease?. Int. J. Epidemiol..

[52] Chang CC (2015). Second-generation PLINK: rising to the challenge of larger and richer datasets. Gigascience.

[53] Zhu Z (2018). Causal associations between risk factors and common diseases inferred from GWAS summary data. Nat. Commun..

[54] Bowden J, Davey Smith G, Burgess S (2015). Mendelian randomization with invalid instruments: effect estimation and bias detection through Egger regression. Int. J. Epidemiol..

[55] Bowden J, Davey Smith G, Haycock PC, Burgess S (2016). Consistent estimation in Mendelian randomization with some invalid instruments using a weighted median estimator. Genet. Epidemiol..

[56] Hartwig FP, Davey Smith G, Bowden J (2017). Robust inference in summary data Mendelian randomization via the zero modal pleiotropy assumption. Int. J. Epidemiol..

[57] Zavos HM (2016). Shared etiology of psychotic experiences and depressive symptoms in adolescence: a longitudinal twin study. Schizophr. Bull..

[58] Chapman LJ, Chapman JP, Kwapil TR, Eckblad M, Zinser MC (1994). Putatively psychosis-prone subjects 10 years later. J. Abnorm. Psychol..

[59] Bogren M (2010). Predictors of psychosis: a 50-year follow-up of the Lundby population. Eur. Arch. Psychiatry Clin. Neurosci..

[60] Liu Y (2011). Meta-analysis of genome-wide association data of bipolar disorder and major depressive disorder. Mol. Psychiatry.

[61] Schizophrenia Psychiatric Genome-Wide Association Study (GWAS) Consortium. (2011). Genome-wide association study identifies five new schizophrenia loci. Nat. Genet..

[62] Muhleisen TW (2014). Genome-wide association study reveals two new risk loci for bipolar disorder. Nat. Commun..

[63] Davies G (2018). Study of 300,486 individuals identifies 148 independent genetic loci influencing general cognitive function. Nat. Commun..

[64] Lee JJ (2018). Gene discovery and polygenic prediction from a genome-wide association study of educational attainment in 1.1 million individuals. Nat. Genet..

[65] den Hoed M (2013). Identification of heart rate-associated loci and their effects on cardiac conduction and rhythm disorders. Nat. Genet..

[66] Kichaev G (2019). Leveraging polygenic functional enrichment to improve GWAS power. Am. J. Hum. Genet..

[67] Astle WJ (2016). The allelic landscape of human blood cell trait variation and links to common complex disease. Cell.

[68] Comuzzie AG (2012). Novel genetic loci identified for the pathophysiology of childhood obesity in the Hispanic population. PLoS ONE.

[69] Michailidou K (2017). Association analysis identifies 65 new breast cancer risk loci. Nature.

[70] van Os J, Linscott RJ, Myin-Germeys I, Delespaul P, Krabbendam L (2009). A systematic review and meta-analysis of the psychosis continuum: evidence for a psychosis proneness-persistence-impairment model of psychotic disorder. Psychol. Med..

[71] Burgess S, Scott RA, Timpson NJ, Davey Smith G, Thompson SG (2015). Using published data in Mendelian randomization: a blueprint for efficient identification of causal risk factors. Eur. J. Epidemiol..

[72] Giusti-Rodriguez P. M. & Sullivan P. F. Using three-dimensional regulatory chromatin interactions from adult and fetal cortex to interpret genetic results for psychiatric disorders and cognitive traits. Preprint at https://www.biorxiv.org/content/10.1101/406330v2 (2019).

[73] Munafo MR, Tilling K, Taylor AE, Evans DM, Davey Smith G (2018). Collider scope: when selection bias can substantially influence observed associations. Int. J. Epidemiol..

[74] Fry A (2017). Comparison of sociodemographic and health-related characteristics of UK Biobank participants with those of the general population. Am. J. Epidemiol..

[75] Taylor AE (2018). Exploring the association of genetic factors with participation in the Avon Longitudinal Study of Parents and Children. Int. J. Epidemiol..

