# Supplementary material for: Genetic overlap between psychotic experiences in the community across age and with psychiatric disorders
Source: Transl Psychiatry. 2020 Mar 9;10:86. doi: 10.1038/s41398-020-0765-2 (PMC7062754; doi:10.1038/s41398-020-0765-2)
Supplement: Supplementary file 1 — Supplementary Figures and Supplementary Note [file 41398_2020_765_MOESM1_ESM.pdf]

Supplementary Figures and Supplementary Note

Genetic overlap between psychotic experiences across age and psychiatric disorders

Table of contents

Page

Figure S1. Manhattan plots for MAGMA gene-based analyses. 2

Figure S2. QQ plots for MAGMA gene-based analyses. 6

Figure S3. Circos plots for chromatin interactions and eQTL. 9

Figure S4. Generalised Summary-Based Mendelian Randomisation analyses. 12

Figure S5. MR-Egger, Weighted Median and Weighted Mode Mendelian randomisation sensitivity analyses. 14

Table S1. Genetic covariance estimates from LD score regression 16

Table S8. MR-Egger intercept test and Cochran Q statistics 17

Table S9. Sensitivity analyses: Mendelian randomization with instrumental variables selected at p < 5 x 10-5 for all exposure measures 18

Extended methods 19

Adolescent psychotic experiences and negative symptom traits 19

Schizotypy during middle adulthood 20

Positive psychotic experiences assessed in adults 20

Mendelian randomization 21

FUMA 22

References 25

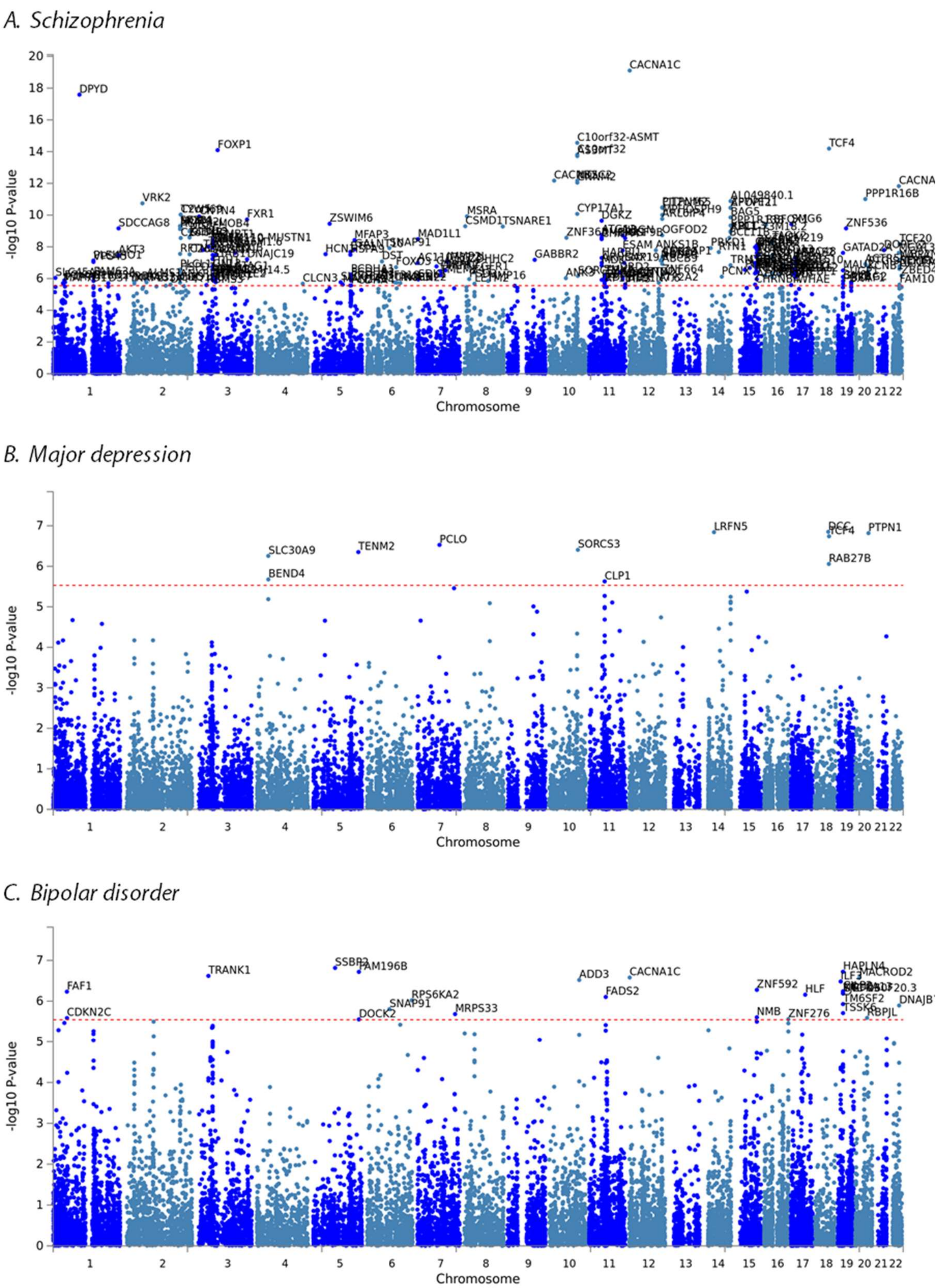

Figure S1. **Manhattan plots for MAGMA gene-based analyses.**

Note: Red dashed line indicates genome-wide significance for schizophrenia as defined at p = 0.05/17674 genes tested = 2.83×10^{-6}, for major depression at p = 0.05/16943 = 2.95×10^{-6} and for bipolar disorder at p = 0.05/17538 = 2.85×10^{-6}.

D. Un-real voice

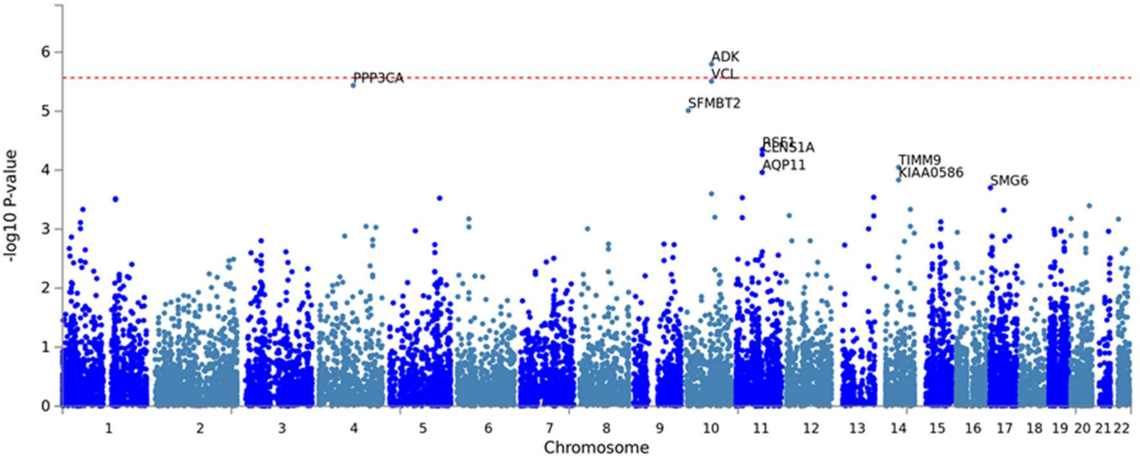

E. Un-real visions

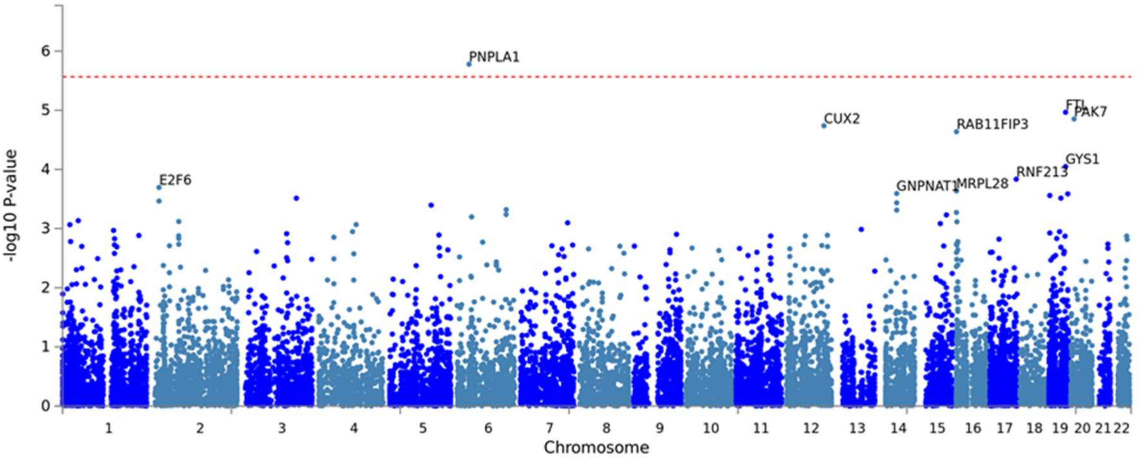

F. Un-real conspiracies

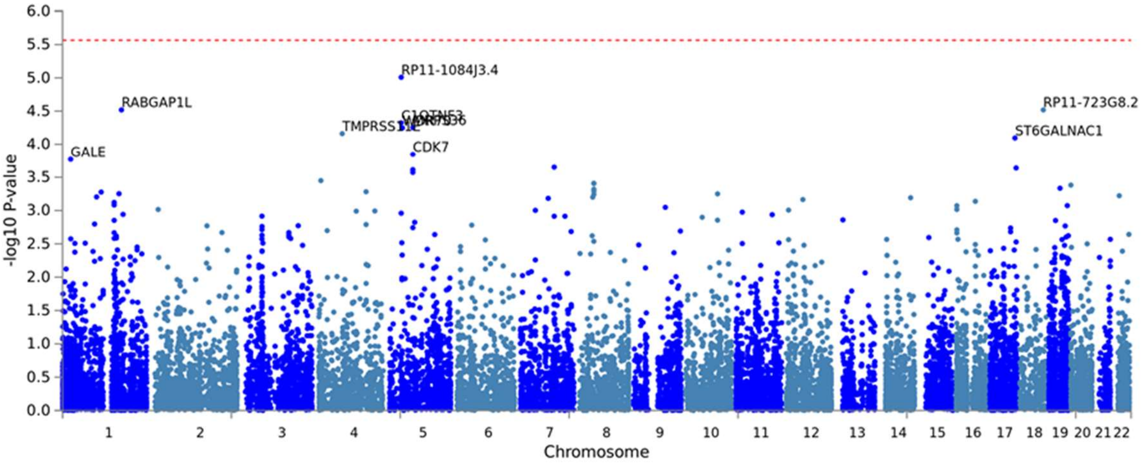

Figure S1. **Manhattan plots for MAGMA gene-based analyses (continued).**

Note: Red dashed line indicates genome-wide significance for positive psychotic experiences in the UK Biobank as defined at p = 0.05/18423 genes tested = 2.71×10^{-6}.

G. Un-real communications

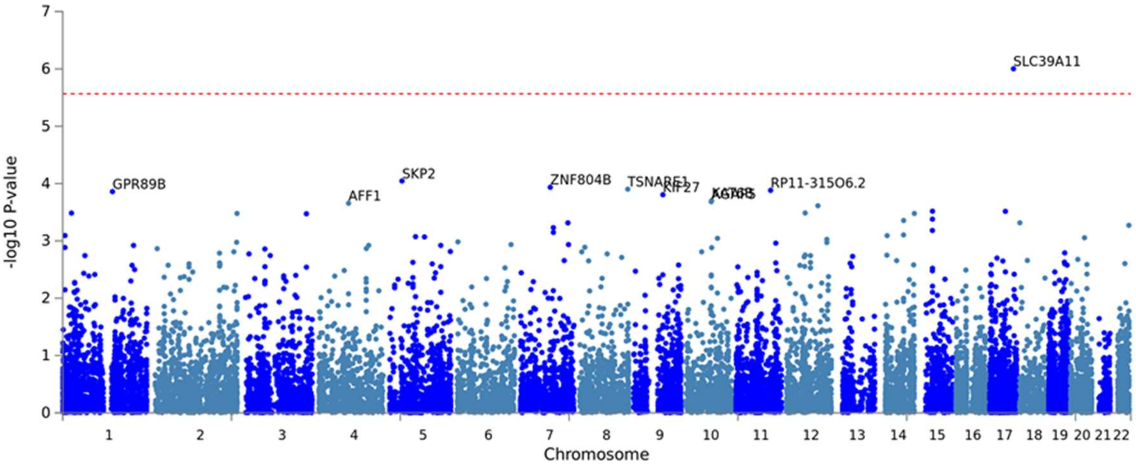

H. Hypomania

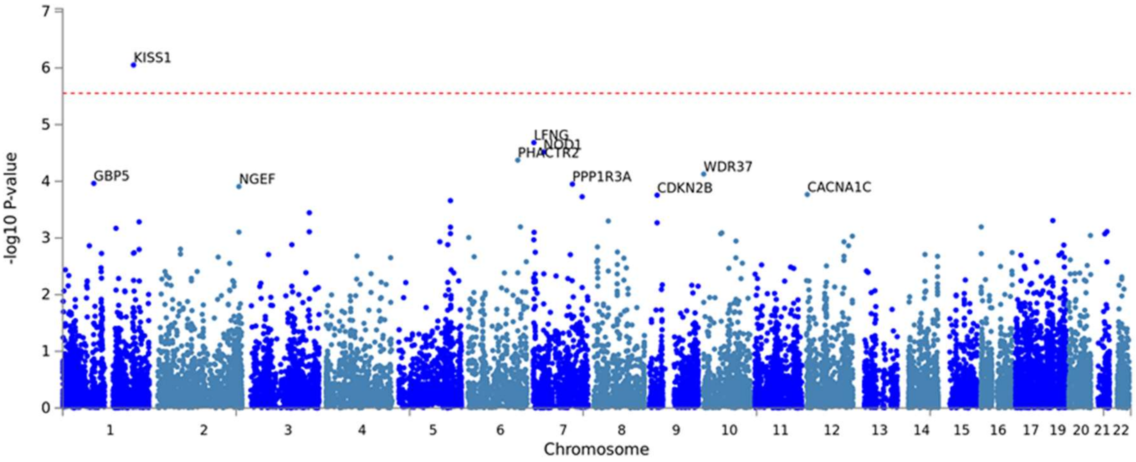

I. Perceptual Aberrations

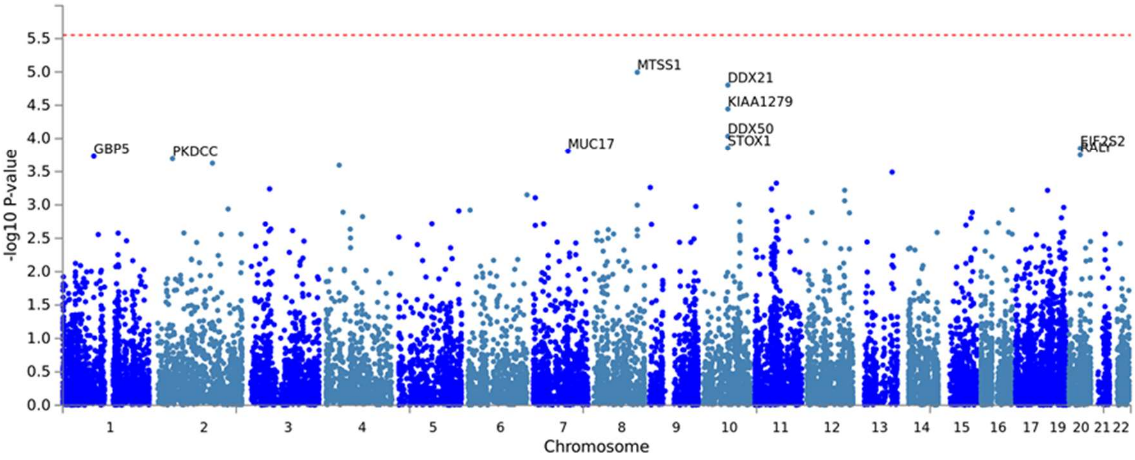

Figure S1. **Manhattan plots for MAGMA gene-based analyses (continued).**

Note: Red dashed line indicates genome-wide significance for positive psychotic experiences in the UK Biobank at defined at P = 0.05/18423 genes tested = 2.714e-6 and for schizotypy in the North Finland Birth Cohort at P = 0.05/17895 = 2.794e-6.

J. Physical anhedonia

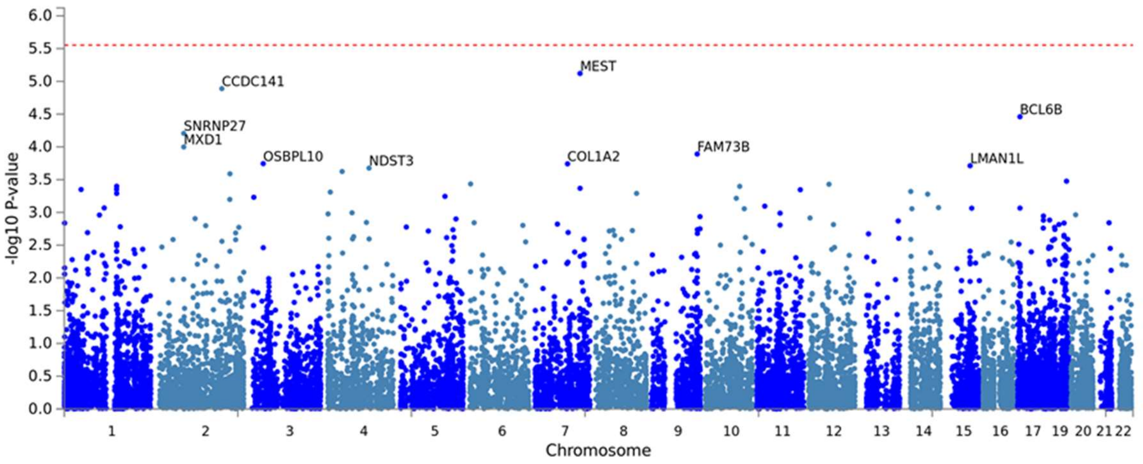

K. Social anhedonia

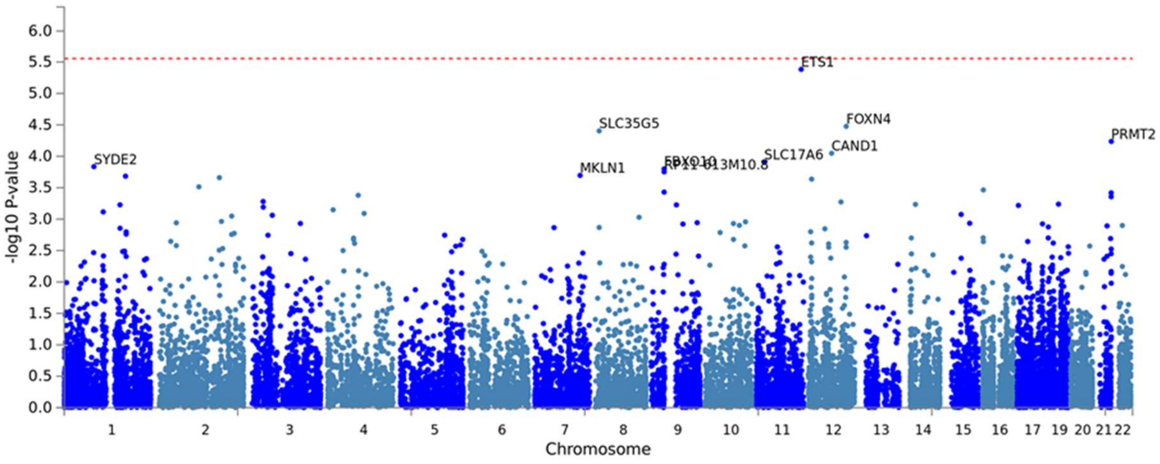

L. Paranoia and hallucinations

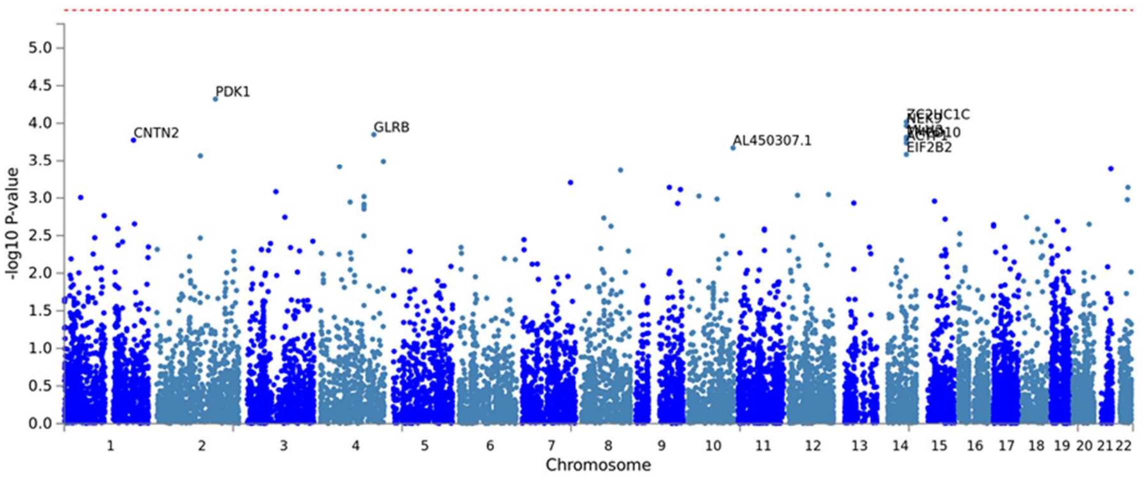

Figure S1. **Manhattan plots for MAGMA gene-based analyses (continued).**

Note: Red dashed line indicates genome-wide significance for schizotypy in the North Finland Birth Cohort at p = 0.05/17895 genes tested = 2.794e-6 and for adolescent psychotic experiences and negative symptom traits at p = 0.05/15957 = 3.133e-6.

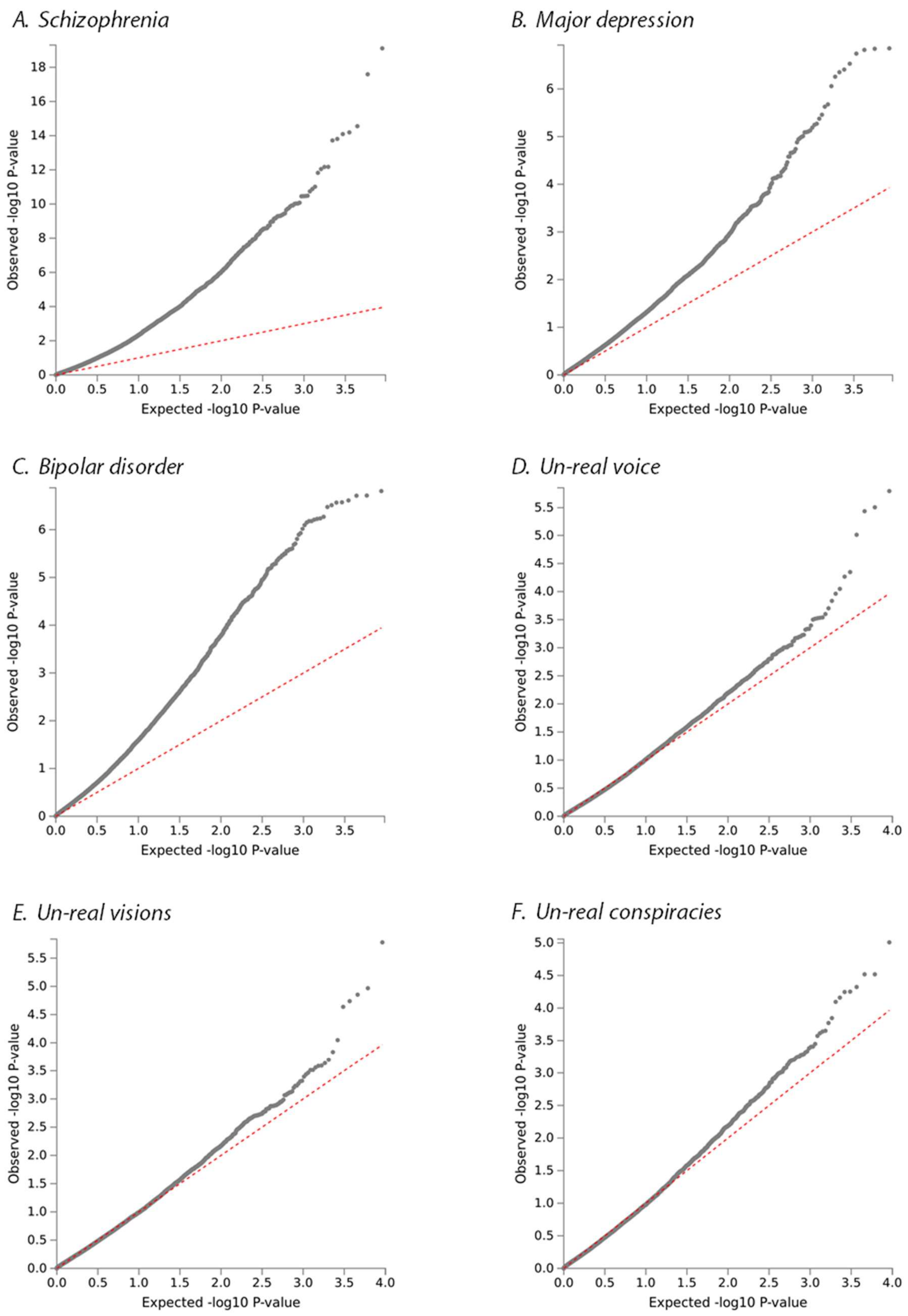

Figure S2. **QQ plots for MAGMA gene-based analyses.**

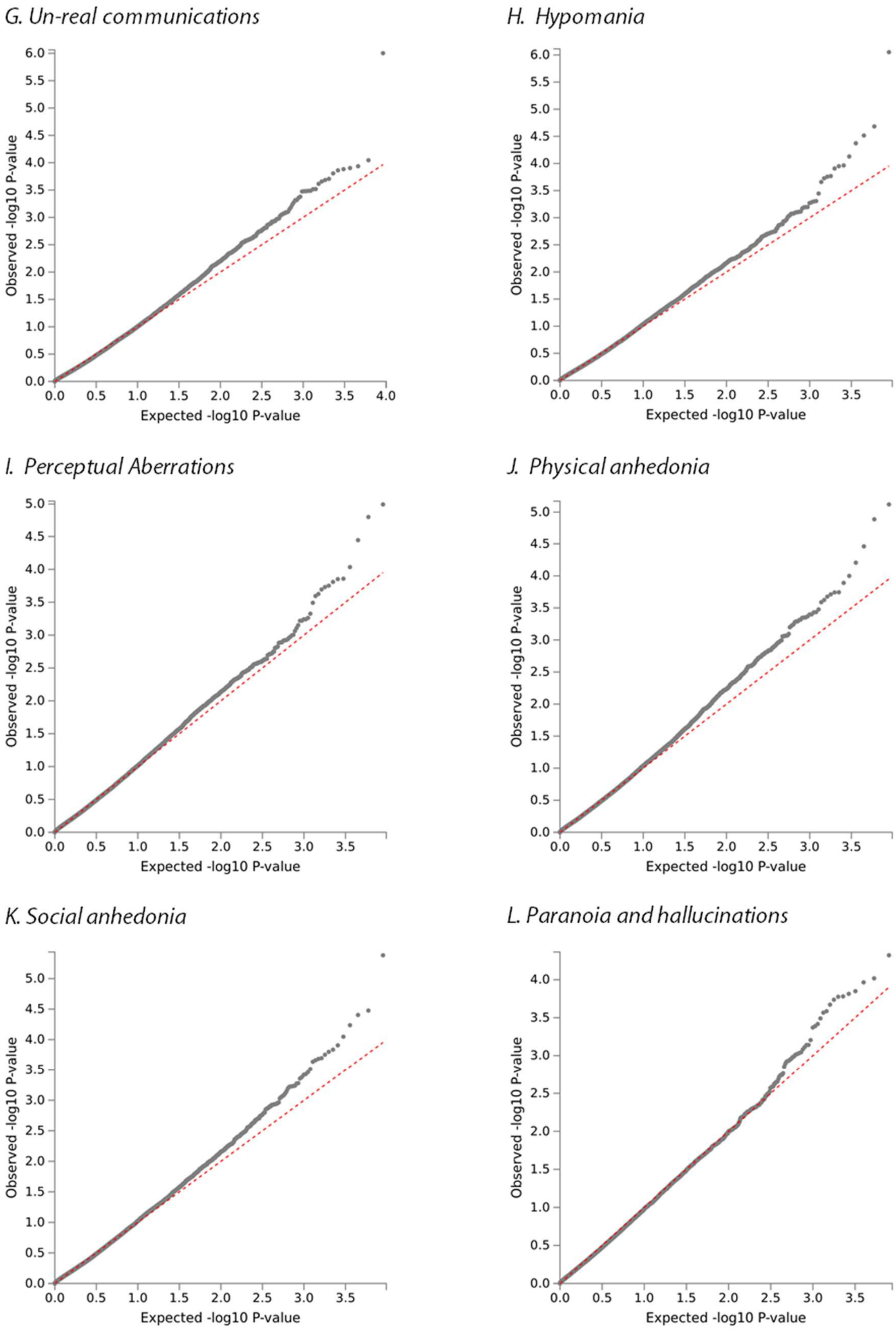

Figure S2. **QQ plots for MAGMA gene-based analyses (continued).**

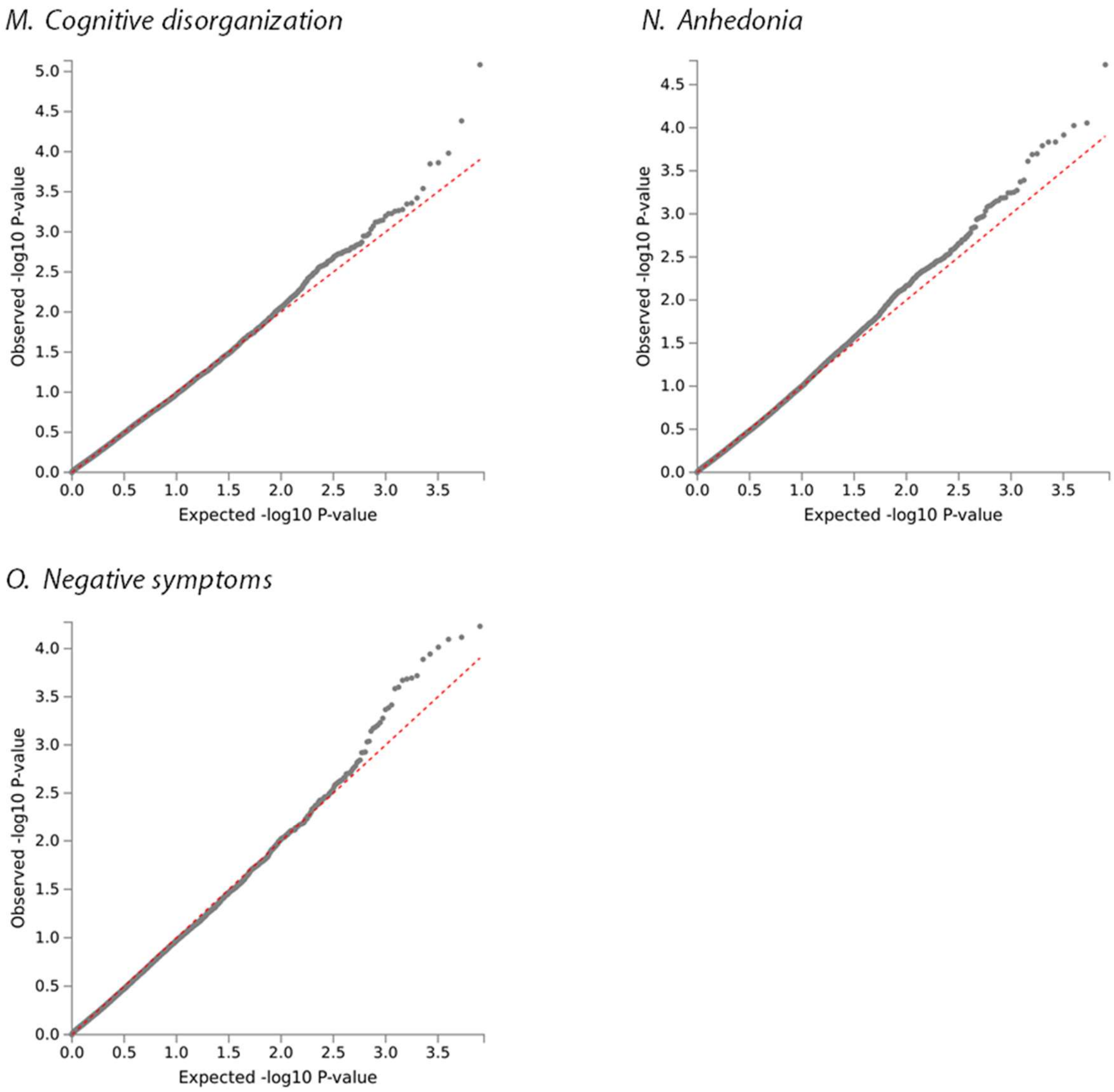

Figure S2. **QQ plots for MAGMA gene-based analyses (continued).**

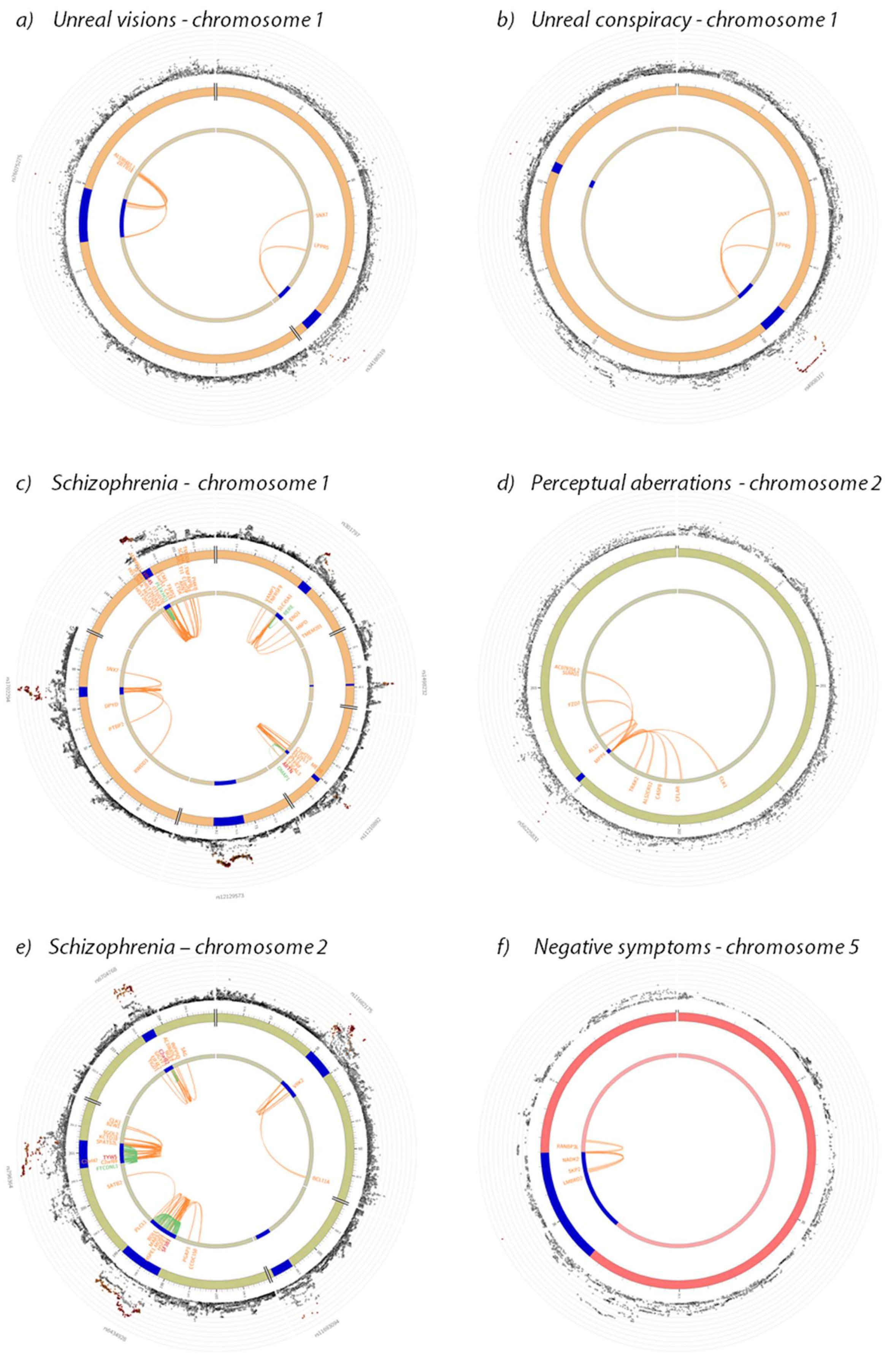

***Figure S3*. Circos plots for chromatin interactions and eQTL.**

g) Unreal communications - chromosome 5

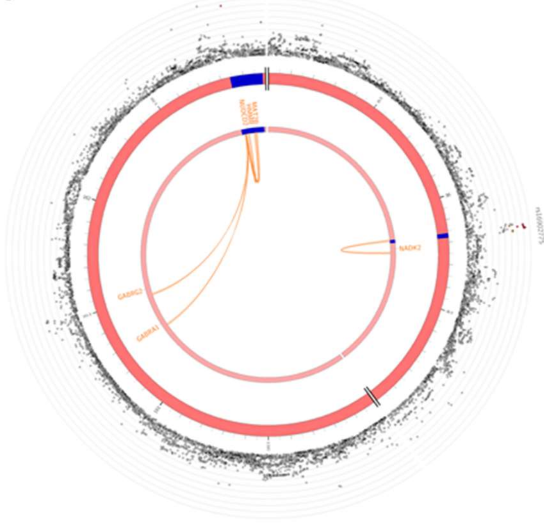

h) Unreal communications – chromosome 8

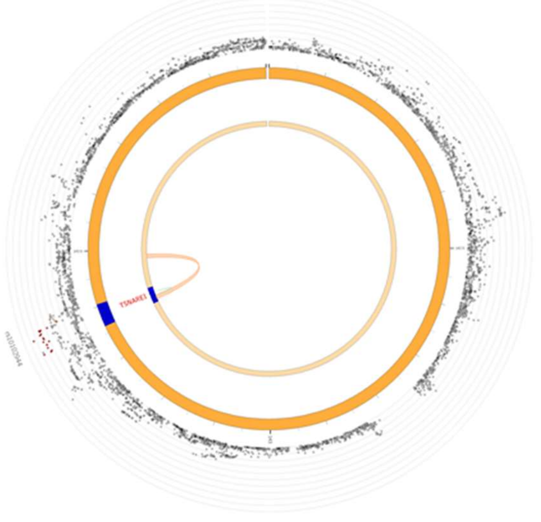

i) Schizophrenia - chromosome 8

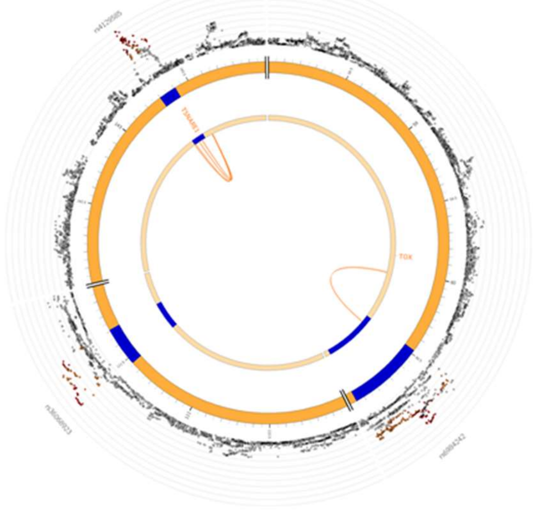

j) Physical anhedonia - chromosome 10

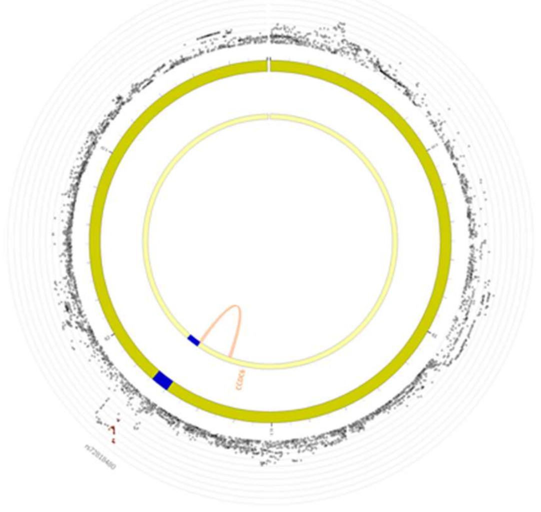

k) Unreal visions - chromosome 10

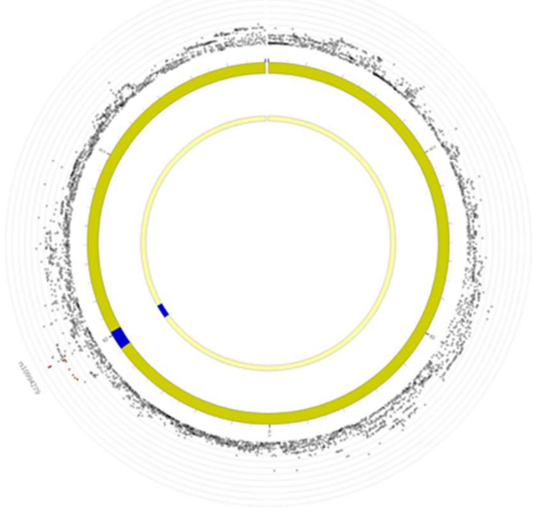

l) Hypomania - chromosome 12

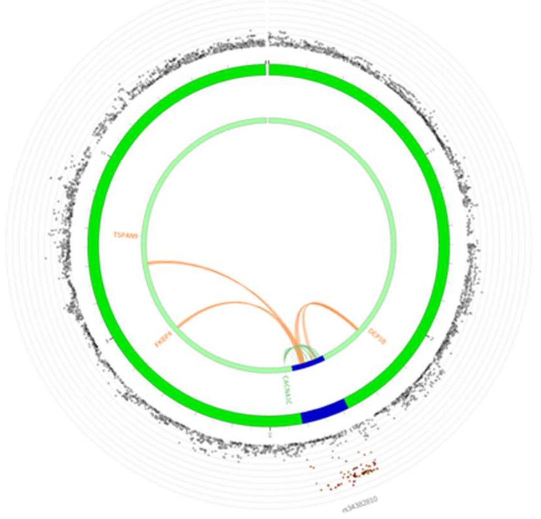

***Figure S3 (continued)*. Circos plots for chromatin interactions and eQTL.**

m) *Unreal visions - chromosome 12*

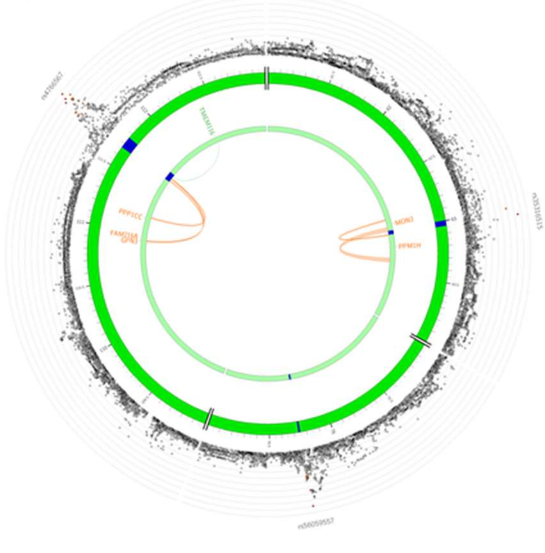

n) *Schizophrenia - chromosome 12*

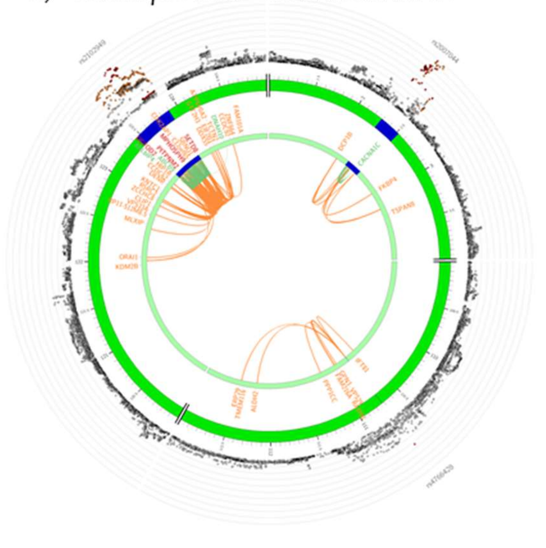

o) *Perceptual aberrations - chromosome 13*

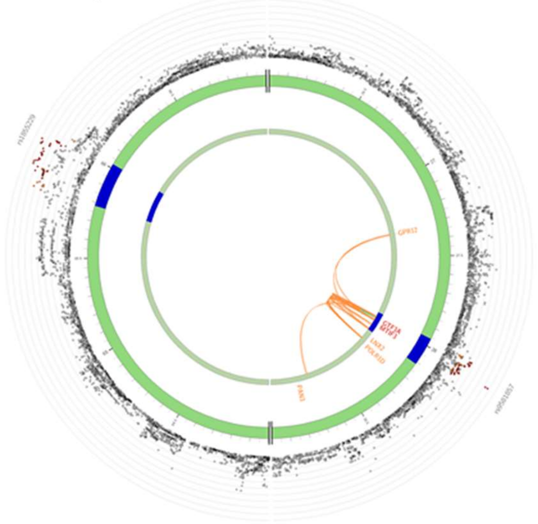

***Figure S3 (continued)*. Circos plots for chromatin interactions and eQTL.**

Note: Manhattan plot displayed in outer most ring with loci colour coded according to the amount of LD shared with lead independent SNPs as follows: red ($r^2 > 0.8$), orange ($r^2 > 0.6$), green ($r^2 > 0.4$) and blue ($r^2 > 0.2$). Genomic risk loci are displayed in blue on the chromosome ring (second and third layers). Genes mapped by chromatin interaction are displayed in orange, by eQTLs in green, and by both chromatin interaction and eQTLs in red; Mapped genes that overlapped between phenotypes are listed in Table 3.

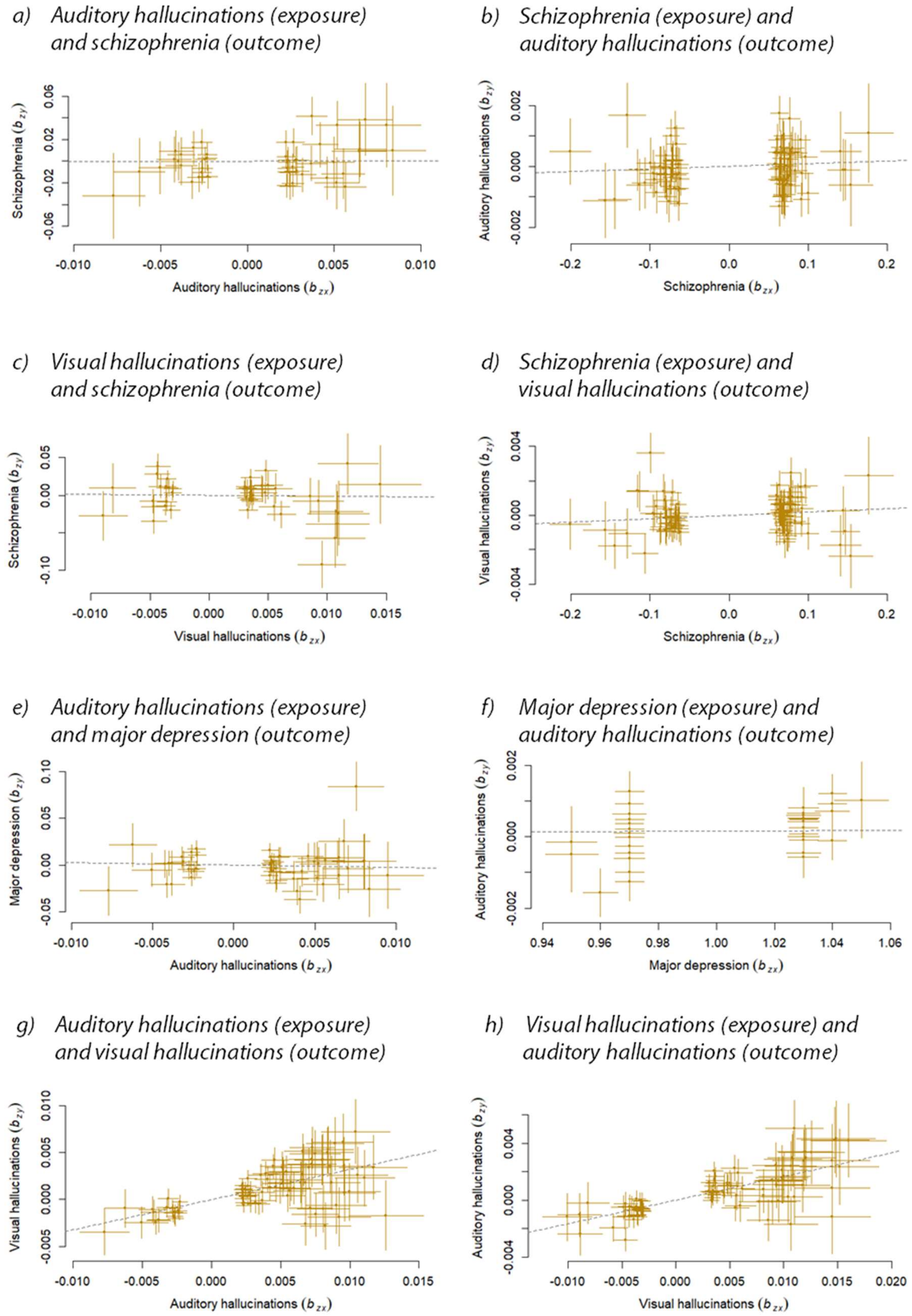

***Figure S4*. Generalised Summary-Based Mendelian Randomisation analyses.**

i) Visual hallucinations (exposure) and major depression (outcome)

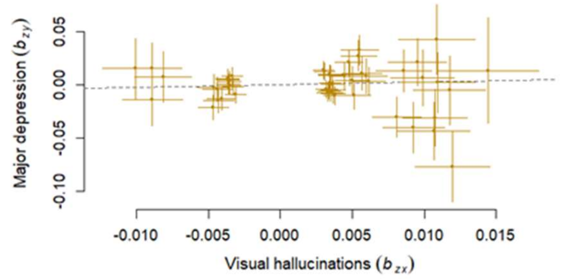

j) Major depression (exposure) and visual hallucinations (outcome)

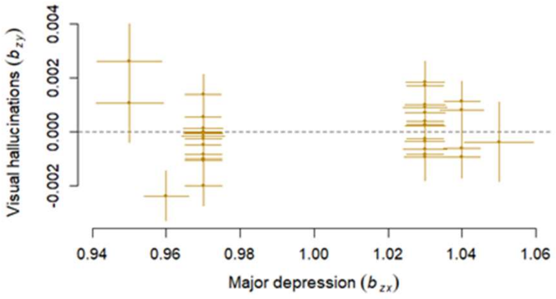

k) Delusions of persecution (exposure) and major depression (outcome)

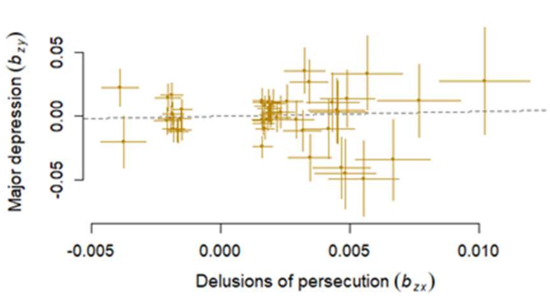

l) Major depression (exposure) and delusions of persecution (outcome)

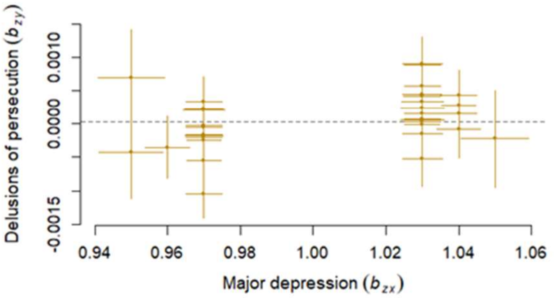

m) Delusions of persecution (exposure) and schizophrenia (outcome)

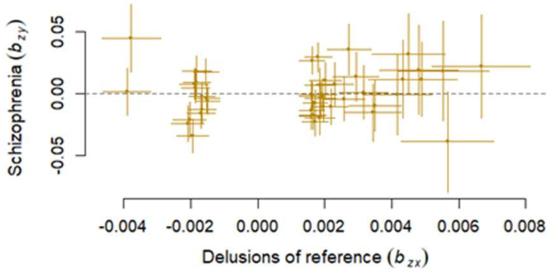

n) Schizophrenia (exposure) and delusions of persecution (outcome)

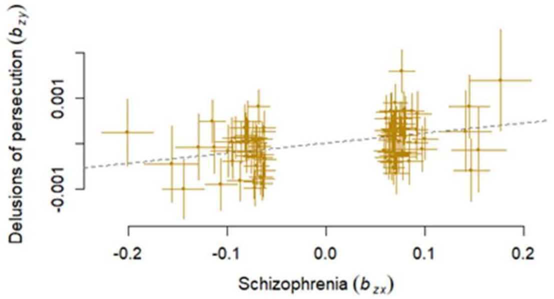

***Figure S4.* Generalised Summary-Based Mendelian Randomisation analyses (continued).**

Note: Scatterplots with the x-axis displaying instrumental variable effects on the exposure (b_{zx}) and the y-axis displaying the instrument-outcome association (b_{zy}). Regression lines included for reference.

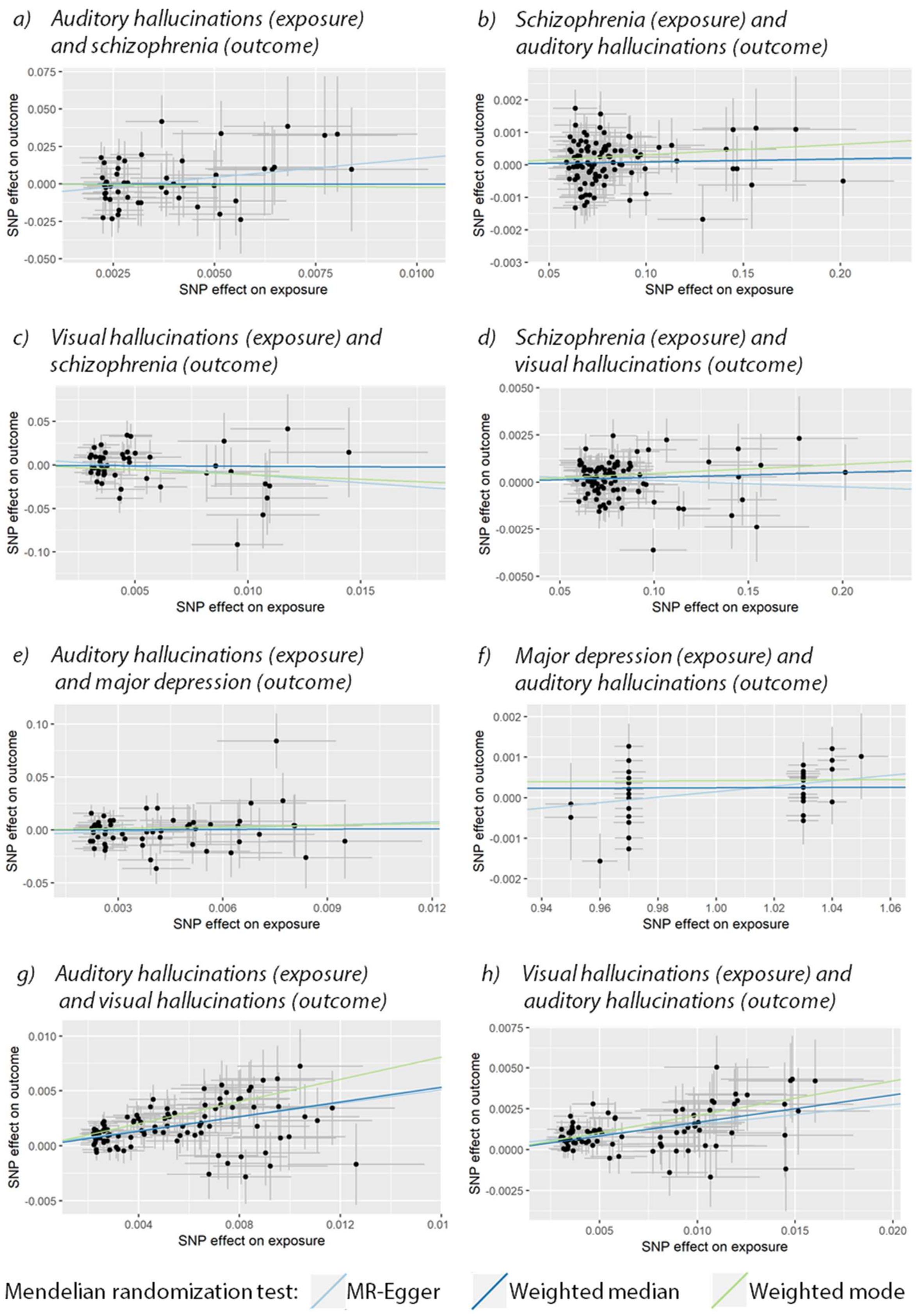

Figure S5. **MR-Egger, Weighted Median and Weighted Mode Mendelian randomisation sensitivity analyses.**

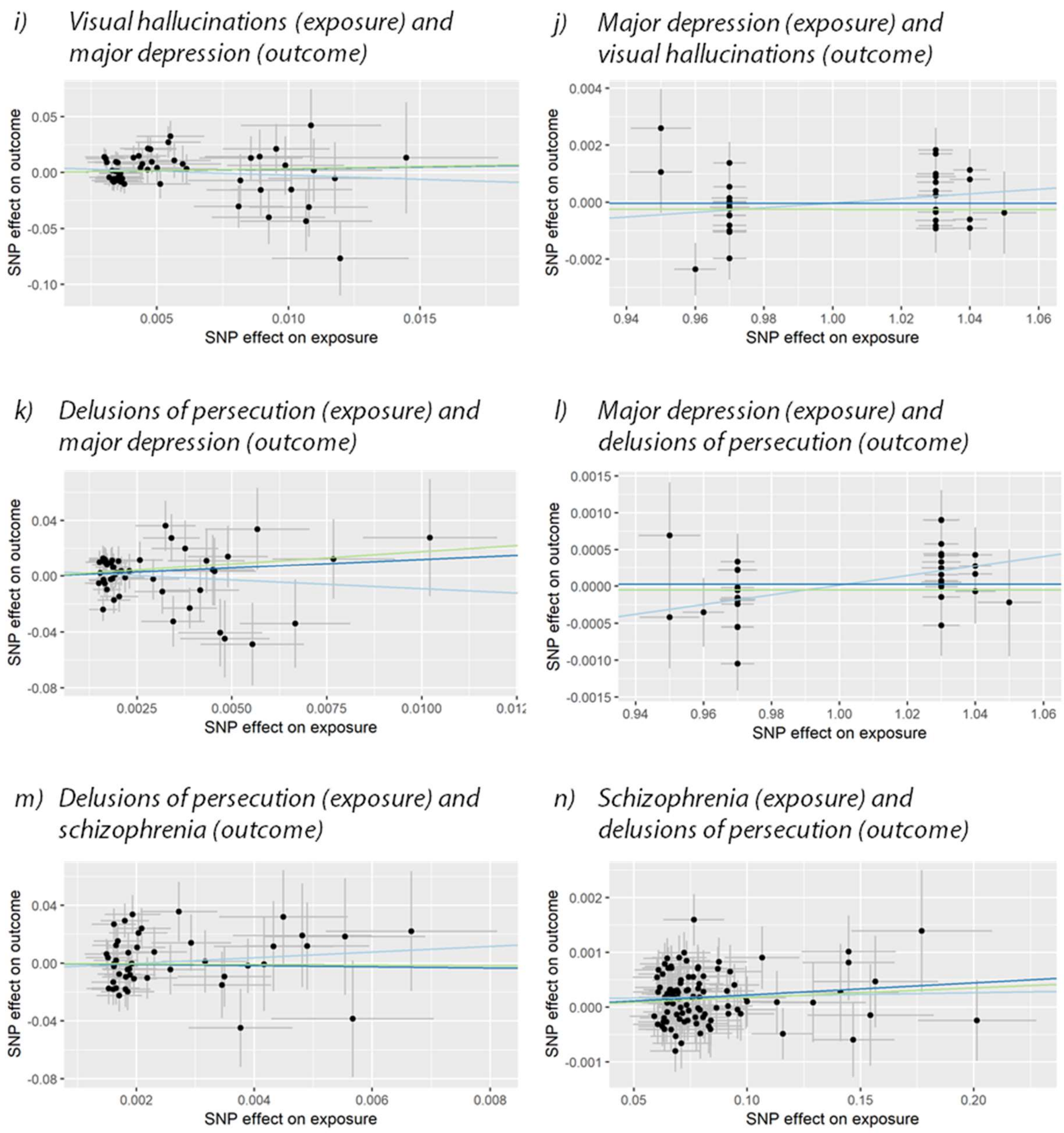

Figure S5. **MR-Egger, Weighted Median and Weighted Mode Mendelian randomisation sensitivity analyses (continued).**

Table S1. **Genetic covariance estimates from LD score regression**

V1	V2	**GenCov intercept (SE)**	**ρ_g**	**SE**	**z1*z2**	**p**
Paranoia and hallucinations	Cognitive disorganisation	0.2361 (0.0065)	0.0053	0.050	0.2370	0.915
Paranoia and hallucinations	Anhedonia	0.1446 (0.0066)	-0.0021	0.048	0.1442	0.965
Paranoia and hallucinations	Negative symptoms	0.0679 (0.0067)	0.0270	0.039	0.0740	0.490
Paranoia and hallucinations	Hypomania	-0.0007 (0.0069)	-0.0183	0.040	-0.0027	0.647
Paranoia and hallucinations	Perceptual aberration	0.0025 (0.0069)	0.0185	0.042	0.0027	0.659
Paranoia and hallucinations	Physical anhedonia	0.0059 (0.0068)	0.0321	0.039	0.0046	0.404
Paranoia and hallucinations	Social anhedonia	0.001 (0.0065)	-0.0177	0.041	-0.0027	0.664
Paranoia and hallucinations	Un-real voice	0.0088 (0.0068)	0.0099	0.008	0.0076	0.193
Paranoia and hallucinations	Un-real vision	0.0011 (0.0072)	0.0123	0.008	0.0093	0.124
Paranoia and hallucinations	Un-real conspiracy	0.002 (0.0064)	0.0120	0.008	0.0093	0.119
Paranoia and hallucinations	Un-real communications	0.0017 (0.0072)	-0.0026	0.007	-0.0020	0.722
Paranoia and hallucinations	Schizophrenia	-0.0044 (0.008)	-0.0009	0.010	0.0003	0.928
Paranoia and hallucinations	Bipolar disorder	0.0017 (0.0078)	0.0002	0.015	0.0022	0.989
Paranoia and hallucinations	Major depression	0.002 (0.0077)	**0.0270**	**0.007**	**0.0257**	**1.43E-04**
Negative symptoms	Cognitive disorganisation	0.1495 (0.0063)	0.0563	0.044	0.1605	0.204
Negative symptoms	Anhedonia	0.1259 (0.0066)	**0.1118**	**0.045**	**0.1480**	**0.012**
Negative symptoms	Hypomania	-0.0056 (0.0074)	0.0159	0.038	0.0026	0.675
Negative symptoms	Perceptual aberration	-0.0021 (0.0063)	0.0360	0.037	0.0058	0.329
Negative symptoms	Physical anhedonia	-0.0014 (0.0069)	-0.0174	0.034	-0.0026	0.603
Negative symptoms	Social anhedonia	-0.0024 (0.0068)	0.0108	0.037	0.0017	0.771
Negative symptoms	Un-real voice	-0.0017 (0.0067)	0.0016	0.007	0.0012	0.827
Negative symptoms	Un-real vision	0.0033 (0.0065)	0.0024	0.007	0.0017	0.732
Negative symptoms	Un-real conspiracy	0.0082 (0.007)	-0.0003	0.006	-0.0003	0.961
Negative symptoms	Un-real communications	0.01 (0.0063)	0.0011	0.007	0.0008	0.875
Negative symptoms	Schizophrenia	-0.0024 (0.0089)	**0.0328**	**0.009**	**0.0281**	**1.37E-04**
Negative symptoms	Bipolar disorder	-0.0091 (0.0073)	0.0099	0.014	0.0045	0.463
Negative symptoms	Major depression	-0.0054 (0.0074)	**0.0346**	**0.007**	**0.0353**	**2.41E-07**
Un-real conspiracy	Un-real communications	0.262 (0.0048)	**0.0091**	**0.003**	**0.2835**	**0.003**

Note: ρ_g = Genetic covariance; GenCov intercept = LD score regression genetic covariance intercept (prior to constraining SNP heritability intercepts) that reflects degree of sample overlap; Genetic covariances are reported in instances where covariance estimates could not be standardised into genetic correlations due to low sample size or SNP heritability. Genetic covariances gives an indication of the presence and direction of genetic overlap but not the magnitude of effect and these results should be interpreted accordingly.

Table S8. **MR-Egger intercept test and Cochran Q statistics**

**Exposure**		**Outcome**	**n SNP**	**MR-Egger**			**MR-Egger intercept test**			**Cochran's Q statistic**		
				**Beta**	**SE**	***p***	**Intercept**	**SE**	***p***	**Q**	**Q_df**	***p***
Un-real voice	→	Schizophrenia	49	2.494	1.996	0.218	-0.008	0.006	0.198	41.57	47	0.696
Schizophrenia	→	Un-real voice	98	0.001	0.004	0.846	0.000	0.000	0.957	104.66	96	0.256
Un-real visions	→	Schizophrenia	48	-1.860	1.697	0.279	0.008	0.007	0.294	61.18	46	0.066
Schizophrenia	→	Un-real visions	96	-0.003	0.005	0.514	0.000	0.000	0.288	97.07	94	0.394
Un-real voice	→	Major depression	55	1.025	1.405	0.469	-0.005	0.005	0.314	62.68	53	0.171
Major depression	→	Un-real voice	36	0.007	0.003	**0.042**	-0.007	0.003	**0.047**	38.23	34	0.283
Un-real voice	→	Un-real visions	97	0.310	0.071	**3.47E-05**	0.000	0.000	0.596	81.86	95	0.830
Un-real visions	→	Un-real voice	85	0.123	0.043	**0.005**	0.000	0.000	0.153	60.93	83	0.967
Un-real visions	→	Major depression	52	-0.711	1.036	0.496	0.005	0.005	0.299	54.88	50	0.295
Major depression	→	Un-real visions	36	0.008	0.005	0.103	-0.008	0.005	0.103	46.74	34	0.072
Un-real conspiracy	→	Major depression	49	-1.264	2.031	0.537	0.004	0.004	0.424	57.13	47	0.148
Major depression	→	Un-real conspiracy	36	0.007	0.002	**0.003**	-0.007	0.002	**0.003**	28.06	34	0.753
Un-real conspiracy	→	Schizophrenia	46	1.976	3.466	0.572	-0.004	0.007	0.576	57.96	44	0.077
Schizophrenia	→	Un-real conspiracy	97	0.001	0.003	0.810	0.000	0.000	0.491	102.81	95	0.274

Note: SNPs excluded during GSMR due to having residual LD and SNPs excluded after Heidi outlier analyses were also excluded from MR-Egger analyses. MR Egger confidence intervals are typically wide but does include the true effect. Generalized Summary-based Mendelian Randomization (GSMR) estimates provides tighter confidence intervals and is more reliable in the absence of horizontal pleiotropy. Horizontal pleiotropy assessed using MR-Egger intercept test where non-significant p-values indicate no evidence of horizontal pleiotropy.

Table S9. **Sensitivity analyses: Mendelian randomization with instrumental variables selected at $p < 5 \times 10^{-5}$ for all exposure measures.**

Exposure		Outcome	Heidi SNPs	LD SNPs	n SNP	GSMR results			MR-Egger			Weighted Median			Weighted Mode		
						Beta	SE	*p*	Beta	SE	*p*	Beta	SE	*p*	Beta	SE	*p*
Auditory hallucinations	→	Schizophrenia	0	0	49	0.021	0.676	0.975	2.494	1.996	0.218	-0.008	0.908	0.993	-0.216	1.993	0.914
Schizophrenia	→	Auditory hallucinations	1	34	886	0.001	0.000	**0.027**	0.001	0.001	0.601	0.001	0.001	0.089	0.004	0.002	0.054
Visual hallucinations	→	Schizophrenia	0	0	48	-0.115	0.501	0.819	-1.860	1.697	0.279	-0.132	0.707	0.852	-1.091	1.635	0.508
Schizophrenia	→	Visual hallucinations	1	34	886	0.002	0.001	**0.001**	0.001	0.002	0.670	0.002	0.001	**0.001**	0.004	0.003	0.179
Auditory hallucinations	→	Major depression	0	0	55	-0.269	0.488	0.581	1.025	1.405	0.469	0.091	0.685	0.895	0.502	1.626	0.759
Major depression	→	Auditory hallucinations	0	9	228	0.004	0.001	**1.17×10^{-5}**	0.003	0.002	0.156	0.004	0.002	0.071	0.005	0.001	**1.01×10^{-6}**
Auditory hallucinations	→	Visual hallucinations	0	0	97	0.321	0.034	**1.73×10^{-21}**	0.310	0.071	**3.47×10^{-5}**	0.333	0.046	**5.16×10^{-13}**	0.505	0.133	**2.66×10^{-4}**
Visual hallucinations	→	Auditory hallucinations	0	0	85	0.166	0.019	**4.25×10^{-18}**	0.123	0.043	**0.005**	0.168	0.026	**1.38×10^{-10}**	0.211	0.065	**1.62×10^{-3}**
Visual hallucinations	→	Major depression	0	0	52	0.263	0.362	0.468	-0.711	1.036	0.496	0.337	0.524	0.520	0.376	1.253	0.765
Major depression	→	Visual hallucinations	0	9	228	0.001	0.001	0.455	0.001	0.003	0.849	0.001	0.003	0.664	0.001	0.001	0.353
Delusions of persecution	→	Major depression	0	0	49	0.356	0.742	0.631	-1.264	2.031	0.537	1.211	1.088	0.266	1.780	2.381	0.458
Major depression	→	Delusions of persecution	0	9	228	0.001	0.001	0.086	-0.001	0.001	0.404	0.000	0.001	0.783	0.001	0.001	0.052
Delusions of persecution	→	Schizophrenia	0	0	46	-0.020	1.025	0.984	1.976	3.466	0.572	-0.401	1.482	0.787	-0.219	2.835	0.939
Schizophrenia	→	Delusions of persecution	0	34	887	0.001	0.000	**2.42×10^{-8}**	0.001	0.001	0.151	0.002	0.000	**2.60×10^{-5}**	0.002	0.002	0.304

Extended methods

Adolescent psychotic experiences and negative symptom traits

The ALSPAC sample (1, 2) invited pregnant women resident in Avon, UK and with an expected delivery date between 1st April 1991 and 31st December 1992 to participate in the study. The initial sample consisted of 14,775 children. Informed consent for the use of data collected via questionnaires and clinics was obtained from participants following the recommendations of the ALSPAC Ethics and Law Committee at the time. Consent for biological samples has been collected in accordance with the Human Tissue Act (2004). Please note that the ALSPAC study website contains details of all the data that is available through a fully searchable data dictionary and variable search tool (http://www.bristol.ac.uk/alspac/researchers/our-data/).

Ethical approval for the original adolescent PENS GWAS (3) was obtained for ALSPAC from the ALSPAC Ethics and Law Committee and the Local Research Ethics Committees, for TEDS from the Institute of Psychiatry ethics committee (ref: 05/Q0706/228), and for CATSS from the Karolinska Institute Ethical Review Board.

The harmonisation process of items for psychotic experiences and negative symptom traits (PENS) across TEDS, CATSS and ALSPAC was informed by principle component analyses, an expert clinical team and the availability of overlapping items (3). For instance, paranoia and hallucinations items from SPEQ that were harmonised across the three adolescent PENS cohorts were "*How often have you thought 'I might be being observed or followed?'*" , for anhedonia "*When something exciting is coming up in my life, I really look forward to it"* (reverse scored), for cognitive disorganisation "*Do you find it difficult in controlling your thoughts?*" and for parent-rated negative symptoms "*My child has a lack of energy and motivation*". Items for anhedonia was not available in the CATSS sample and for cognitive disorganisation unavailable in ALSPAC.

Linear regression GWAS and Generalized Estimating Equation (GEE; to account for the presence of twin pairs) was performed on the four PENS scales (3). Summary result files were obtained from the authors with permission from the original study cohorts.

Schizotypy during middle adulthood

Four schizotypy scales were used to assess psychotic experiences during middle adulthood: Perceptual aberrations were assessed with the Perceptual Aberration Scale (4) with 35 true/false items devised to assess experiences in the general population that resemble clinical features of schizophrenia with an emphasis on body image aberrations including unclear body boundaries, body size and physical attributes being distorted, or feelings of estrangement from one's own body. Items also assessed unusual visual and auditory experiences, for example "*My hearing is sometimes so sensitive that ordinary sounds become uncomfortable*" and "*Sometimes when I look at things like tables and chairs, they seem strange*".

Hypomania was from the Hypomanic Personality Scale (5) and consisted of 48 true/false items devised to assess hypomania, gregariousness, grandiosity and euphoria (e.g. "*I can usually slow myself down when I want to"* and "*I have often been so excited about an involving project that I didn't care about eating or sleeping*").

Two scales from Chapman's Schizotypia Scales were employed to assess social anhedonia with the Revised Social Anhedonia Scale and physical anhedonia from the Revised Physical Anhedonia Scale (6), devised to assess the inability to take pleasure from physical (61 true/false items, e.g. "*One food tastes as good as another to me*") and social (40 true/false items, e.g. *"I prefer watching television to going out with other people"*) stimuli respectively.

Summary statistics from linear regression GWAS performed on these four schizotypy scales were obtained from the authors (7).

Positive psychotic experiences assessed in adults

GWAS on four dichotomous items from the UK Biobank were included. The items assessed psychotic experiences in adults aged 40-69 years: Whether participants ever experienced auditory hallucinations (UK Biobank phenotype ID = 20463; *"Did you ever hear things that other people said did not exist, like strange voices coming from inside your head talking to you or about you, or voices coming out of the air when there was no one around?"*), visual

hallucinations (UK Biobank phenotype ID = 20471; *"Did you ever see something that wasn't really there that other people could not see?"*), delusions of persecution (UK Biobank phenotype ID = 20468; *"Did you ever believe that there was an unjust plot going on to harm you or to have people follow you, and which your family and friends did not believe existed?"*) and experienced delusions of reference (UK Biobank phenotype ID = 20474; *"Did you ever believe that a strange force was trying to communicate directly with you by sending special signs or signals that you could understand but that no one else could understand (for example through the radio or television)?"*). The mean age of onset of positive psychotic experiences reported by UK Biobank participants was 31.6 (s.d. = 17.6) years. Of those reporting positive psychotic experiences, 11.3% indicated that they have received medication for psychotic experiences and 21.3% have talked to a mental health professional about their psychotic experiences.

Separate linear regression GWAS was performed on each of the four positive psychotic experiences items for individuals of European ancestry (N = 116,787 - 117,794) by Neale Lab and summary results was downloaded from the Neale Lab website (http://www.nealelab.is/uk-biobank).

Mendelian randomization

Mendelian randomization (MR)(8) was conducted to further explore the relationship between psychotic experiences and psychiatric disorders for the phenotype pairs that had significant genetic correlations. MR is used to test for a causal relationship between an exposure (the putatively causal trait) and outcome trait by using instrumental variables as proxies for the exposure trait. In MR, instrumental variables are SNPs robustly associated with the exposure based on GWAS results. Due to the random nature of Mendelian segregation of genetic variants during meiosis, the extent to which unmeasured confounding factors influence the outcome is not expected to differ between those who inherited a specific copy of a genetic variant and those who did not (akin to the randomization process employed in randomized controlled trials).

The presence of a causal association between the exposure (X) on the outcome (Y) trait can be calculated as the ratio of the effect size of a SNP instrumental variable (Z) on the

outcome over its effect on the exposure: $\hat{b}_{XY} = \hat{b}_{ZY}/\hat{b}_{ZX}$, where $\hat{b}_{XY}$ is the effect of the exposure on the outcome, $\hat{b}_{ZY}$ is the effect of the SNP instrument variable on the outcome and $\hat{b}_{ZX}$ is its effect on the exposure. To overcome the small effect sizes of individual SNPs, multiple SNPs are used as instrumental variables to increase power and an aggregate $\hat{b}_{XY}$ effect can be obtained using a generalized least squares approach (9).

The effect alleles for summary statistics for positive psychotic experiences, schizophrenia and major depression were harmonised to be in phase with the 1000 Genomes (phase 3) reference panel in both the outcome and exposure data. The effects were log odds ratios for binary traits, except for the UK Biobank psychotic experiences for which linear regression coefficients were provided.

FUMA

FUMA is a web application that offers a streamlined pipeline to perform several post-GWAS analyses on summary statistics. Summary statistics are uploaded to a server and automatically deleted once the analyses have been performed. Results are stored on the FUMA servers until users remove these.

Post-GWAS functional annotation analyses for adolescent PENS and adult schizotypy was reported in the original GWAS publications (3, 7) but not for positive psychotic experiences in the UK Biobank. To aid the comparison between the three psychotic experiences cohorts, we performed SNP annotations and gene mapping analyses on all psychotic experiences summary statistics using the same quality control procedures, methods and parameters within the FUMA pipeline (10) as follows: LD independent lead SNPs were identified at $p < 1 \times 10^{-5}$ for PE, at $p < 1 \times 10^{-6}$ for MDD, and at $p < 1 \times 10^{-8}$ for schizophrenia and bipolar disorder (p-value thresholds were set to allow for more than 20 independent SNPs to be analysed) within a 250kb window at $r^2 < 0.1$ based on LD structure in the 1000 Genomes phase 3 reference panel for individuals of European decent.

Annotation of functional consequences associated with independent lead SNPs and SNPs obtained from the reference panel that are in LD with independent SNPs (at $r^2 \geq 0.6$) was performed using ANNOVAR (11) (based on Ensembl genes build version 92) whilst

excluding the extended MHC region (25,000,000-35,000,000). ANNOVAR is a software tool used to identify whether SNPs are associated with protein coding or amino acid changes. Annotation are based on several sources of information such as gene or splicing site locations, mRNA sites, genomic region-based information such as conserved regions and predicted transcription factor binding sites, stable RNA secondary structures or microRNA target sites. ANNOVAR offers the utility to use several public databases for a range of functional annotations as well as options on which to filter variants, such as SIFT scores for non-synonymous mutations. Based on user-defined gene definition databases like Ensembl, ANNOVAR annotates each variant to indicate its position in relation to genes (for instance, whether the variant is exonic, intronic, within a splicing site, upstream or downstream from a gene). For non-synonymous single nucleotide variants or indels, amino acid changes are also annotated. Precomputed functional importance scores, such as CADD scores (12), that indicate how likely a variant would have deleterious consequences, can also be annotated to variants. Based on these variant annotations, ANNOVAR offers the option to automate the process of gene mapping according to user-defined parameters.

Mapping of variants to the most likely causal genes was performed by employing a combination of positional mapping, expression quantitative trait loci (eQTL) mapping and 3D chromatin interaction mapping using the following parameters. Gene mapping was performed on lead independent SNPs and SNPs from the 1000 Genomes reference panel for individuals of European descent that were in LD with lead SNPs at $r^2 > 0.6$. For positional mapping, variants located within 10kb of known gene regions were mapped to genes if likely to be deleterious based on a CADD score ≥ 12.37 (12). eQTL mapping of SNPs to genes were performed based on significant eQTL associations at a false discovery rate (FDR) < 0.05 obtained from 13 brain regions from GTEx v7 brain tissue repository and 10 from GTEx v6 (13, 14). SNPs were mapped to genes based on significant chromatin interactions obtained from high-resolution HiC datasets for fetal and adult human brain samples (15) and for the dorsolateral prefrontal cortex and the hippocampus from GSE87112 at the recommended FDR of $p < 1 \times 10^{-6}$ 250kb upstream and 500kb downstream from the transcription start site (16). Promoter and enhancer regions were annotated from the Roadmap 111 epigenomes brain tissue for 13 brain regions (17, 18). Additionally, parameters in FUMA was set to map variants within protein-coding regions only.

Note that full gene mapping results from FUMA for each phenotype are provided in Tables S2 – 7 as additional data files and are available online.

References

1. Boyd A, Golding J, Macleod J, Lawlor DA, Fraser A, Henderson J, et al. (2013): Cohort Profile: the 'children of the 90s'--the index offspring of the Avon Longitudinal Study of Parents and Children. *Int J Epidemiol*. 42:111-127.
2. Fraser A, Macdonald-Wallis C, Tilling K, Boyd A, Golding J, Davey Smith G, et al. (2013): Cohort Profile: the Avon Longitudinal Study of Parents and Children: ALSPAC mothers cohort. I*nt J Epidemiol*. 42:97-110.
3. Pain O, Dudbridge F, Cardno AG, Freeman D, Lu Y, Lundstrom S, et al. (2018): Genome-wide analysis of adolescent psychotic-like experiences shows genetic overlap with psychiatric disorders. *Am J Med Genet B Neuropsychiatr Genet*. 177:416-425.
4. Chapman LJ, Chapman JP, Raulin ML (1978): Body-image aberration in Schizophrenia. *J Abnorm Psychol*. 87:399-407.
5. Eckblad M, Chapman LJ (1986): Development and validation of a scale for hypomanic personality. *J Abnorm Psychol*. 95:214-222.
6. Chapman LJ, Chapman JP, Raulin ML (1976): Scales for physical and social anhedonia. *J Abnorm Psychol*. 85:374-382.
7. Ortega-Alonso A, Ekelund J, Sarin AP, Miettunen J, Veijola J, Jarvelin MR, et al. (2017): Genome-Wide Association Study of Psychosis Proneness in the Finnish Population. *Schizophr Bull*. 43:1304-1314.
8. Davey Smith G, Ebrahim S (2003): 'Mendelian randomization': can genetic epidemiology contribute to understanding environmental determinants of disease? *Int J Epidemiol*. 32:1-22.
9. Zhu Z, Zheng Z, Zhang F, Wu Y, Trzaskowski M, Maier R, et al. (2018): Causal associations between risk factors and common diseases inferred from GWAS summary data. *Nat commun*. 9:224.
10. Watanabe K, Taskesen E, van Bochoven A, Posthuma D (2017): Functional mapping and annotation of genetic associations with FUMA. *Nat commun*. 8:1826.
11. Wang K, Li M, Hakonarson H (2010): ANNOVAR: functional annotation of genetic variants from high-throughput sequencing data. *Nucleic Acids Res*. 38:e164.

12. Kircher M, Witten DM, Jain P, O'Roak BJ, Cooper GM, Shendure J (2014): A general framework for estimating the relative pathogenicity of human genetic variants. *Nat genet*. 46:310-315.

13. GTEx Consortium (2015): Human genomics. The Genotype-Tissue Expression (GTEx) pilot analysis: multitissue gene regulation in humans. *Science*. 348:648-660.

14. GTEx Consortium, Battle A, Brown CD, Engelhardt BE, Montgomery SB (2017): Genetic effects on gene expression across human tissues. *Nature*. 550:204-213.

15. Giusti-Rodriguez PM, Sullivan PF (2019): Using three-dimensional regulatory chromatin interactions from adult and fetal cortex to interpret genetic results for psychiatric disorders and cognitive traits. *bioRxiv*. 406330.

16. Schmitt AD, Hu M, Jung I, Xu Z, Qiu Y, Tan CL, et al. (2016): A Compendium of Chromatin Contact Maps Reveals Spatially Active Regions in the Human Genome. *Cell Rep*. 17:2042-2059.

17. Kundaje A, Meuleman W, Ernst J, Bilenky M, Yen A, Heravi-Moussavi A, et al. (2015): Integrative analysis of 111 reference human epigenomes. *Nature*. 518:317-330.

18. Ernst J, Kellis M (2012): ChromHMM: automating chromatin-state discovery and characterization. *Nat Methods*. 9:215-216.
